# Reachability bounds for chemical reaction networks and strand displacement systems

**DOI:** 10.1007/s11047-013-9403-8

**Published:** 2013-12-22

**Authors:** Anne Condon, Bonnie Kirkpatrick, Ján Maňuch

**Affiliations:** 1 Department of Computer Science, University of British Columbia, Vancouver, BC Canada; 2 Department of Mathematics, Simon Fraser University, Burnaby, BC Canada

**Keywords:** Chemical reaction networks, Strand displacement systems, Reachability bounds

## Abstract

Chemical reaction networks (CRNs) and DNA strand displacement systems (DSDs) are widely-studied and useful models of molecular programming. However, in order for some DSDs in the literature to behave in an expected manner, the initial number of copies of some reagents is required to be fixed. In this paper we show that, when multiple copies of all initial molecules are present, general types of CRNs and DSDs fail to work correctly if the length of the shortest sequence of reactions needed to produce any given molecule exceeds a threshold that grows polynomially with attributes of the system.

## Introduction

DNA strand displacement systems (DSDs) (Yurke and Mills 2003; Zhang et al. 2007) and chemical reaction networks (CRNs) (Cook et al. 2009; Soloveichik 2009, 2008) are important molecular programming models. DSDs provide sophisticated molecular realizations of logic circuits and even artificial neurons (Qian and Winfree 2011; Qian et al. 2011b), while CRNs elegantly express chemical programs that can then be translated into DSDs (Chen et al. 2012; Soloveichik et al. 2008, 2010). CRNs and thus DSDs can in principle simulate Turing-general models of computation (Qian et al. 2011a; Seelig et al. 2006), and DSDs can be energy efficient (Seelig et al. 2006; Soloveichik et al. 2010; Yurke et al. 2000; Zhang and Seelig 2011). It is also possible in principle to recycle molecules in DSDs by running reversible reactions or displacements in both forwards and reverse directions, so that *t* steps of the system use just *O*(log *t*) molecules (Condon et al. 2012; Thachuk and Condon 2012).

However, correct behavior of some published DSDs (Condon et al. 2012; Qian et al. 2011a) requires that an exact numbers of some reactants are present initially, and it is currently impractical to obtain the exact numbers in a wet lab. We previously considered the conditions for a class of CRNs to work correctly when multiple copies of all initial molecules are present and showed that the length of the shortest trace (sequence of reactions) needed to “reach”, i.e., produce, any given molecule is bounded by a polynomial function of some attributes of a CRN in this class (Condon et al. 2012). This reachability upper bound reveals important limits of molecular programs that fall in the class covered by our result: we cannot write such programs that run correctly in a closed chemical system and for which the number of steps (reactions) of the program is sufficiently large relative to the volume of initial reagents. 1

In this work we provide two new reachability upper bounds that significantly extend our earlier work. The first new theorem applies to *tagged* CRNs which, as we explain below, are important because they can be translated into DSDs of comparable volume that can simulate the CRN traces. The second new theorem applies to a broader class of DSDs than does the translated version of our first result. In the rest of this introduction we motivate our results in more detail. Sections 2 and 3 provide technical details of both theorems. We list some open questions in Sect. 4.

### New result for chemical reaction networks (CRNs)

**Fig. 1:**
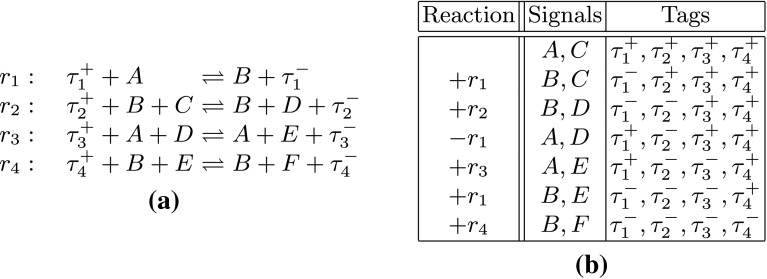
Example of a simple tagged chemical reaction network (CRN).** a** List of reactions, all of which are reversible.** b** Changes in signal and tag species as reactions occur. The first row lists species present initially. The left column of subsequent rows lists the reaction applied, with* plus* indicating the forward and* minus* indicating the backwards direction. Two right columns show the signal and tag species, respectively, after the reaction has been applied

Figure 1a illustrates a CRN of the type to which our new result applies (a formal definition of CRN is in Sect. 2). Each reaction *r*_*i*_ is reversible, and has unique *tag* species* τ*_*i*_^+^ and* τ*_*i*_^−^ on its left and right sides respectively. We explain later why we focus on tagged CRNs, and also explain why we ignore reaction rate constants in our example and results.

When a single copy of each species in the set {*A*, *C*, *τ*_1_^+^, *τ*_2_^+^, *τ*_3_^+^, *τ*_4_^+^} is initially present, it takes six reaction steps to produce the product *F*, and to do so, reaction *r*_1_ must run in the forwards direction, then later run backwards, then forwards again, cf. Fig. 1b. However, if another copy of *A* is present initially then *F* can be generated with just four reactions. The behavior of the system with two copies does not mirror its behavior with one copy; in this sense it is incorrect. While for this simple example it might not seem important how many steps are needed to produce a particular product, it is critically important in contexts where the product is the result of a computation and an erroneous result could be produced as a result of cross-talk, or short-circuiting of multiple copies of the intended computation.

In this paper, our notion of correctness is that of *copy tolerance* (Condon et al. 2012). We say that a CRN **C** is *x*-copy-tolerant if the length of the shortest trace that produces any species *s* in **C** and in **C** ^(*x*)^ is the same, where **C** ^(*x*)^ is the CRN with the same reactions as **C** but with *x* initial copies of each initial molecule of **C**. A system is copy-tolerant if it is *x*-copy-tolerant for all *x*. The CRN of Fig. 1 is not 2-copy-tolerant. Copy-tolerance is a weak notion of correctness; if a CRN **C** is not 2-copy tolerant then, for example, **C** also fails to satisfy the stronger requirement that each possible trace of **C** in the 2-copy setting is an interleaving of two possible traces in the single copy setting. We chose to work with a weak notion of correctness because it makes our results stronger, i.e., they apply also to notions of correctness that are stronger than copy-tolerance.

Our first reachability upper bound, Theorem 2, shows that in order for a tagged CRN **C** to be copy-tolerant, the number of steps needed for **C** to produce any given species must be suitably bounded. The bound is a polynomial function of the volume and other attributes of **C**.

We prove our result for *tagged* CRNs—CRNs with a unique species on the left and right side of each reaction (Fig. 1)—for two reasons. First, the tags make it possible for us to prove strong results. The second reason stems from the fact that our ultimate goal is to prove limits on the power of DSDs, which can be realized with DNA strands, rather than for CRNs which are a useful theoretical abstraction. When translating an “untagged” CRN to a DSD, two sets of auxiliary DNA strand complexes, which we refer to as transformers, are introduced per reaction of the CRN, one set for each side of the reaction. Each set of transformers includes unique strands that do not otherwise appear in the DSD. The CRN tag species represent the sets of transformer DNA strands. Put another way, to translate an untagged CRN to a DSD using current methods, it is necessary to first add tags to the CRN and then map the tags to the sets of transformer species. Thus, by proving a reachability upper bound for a tagged CRN, we are obtaining a result for the DSD realization of the corresponding untagged CRN. The result would apply also to other realizations of CRNs, perhaps even using molecules other than DNA, in which transformer molecules are needed in the realization. Our earlier result (Condon et al. 2012) did not apply to general tagged CRNs.

Unlike the example of Fig. 1, chemical reactions have associated kinetic rate constants that, along with species counts, determine reaction propensities (Soloveichik 2009; Soloveichik et al. 2008). In particular, a CRN behaves stochastically if multiple reactions are applicable to the molecules available at one or more points in the sequence of reactions. However, in examples such as the stack machine of Qian et al. (2011a) and the Gray code counter of Condon et al. (2012), correctness of the CRN does not depend on the relative propensities of applicable reactions (although the expected time to complete the simulation of the CRN does depend on those propensities). Since our results are expressed in terms of number of reactions rather than reaction propensities, they apply to stochastic CRNs. We can interpret our reachability result as a hitting time in the stochastic context where a hitting time is the minimum number of reactions required to reach a goal state from the initial state.

### New result for strand displacement systems (DSDs)

**Fig. 2:**
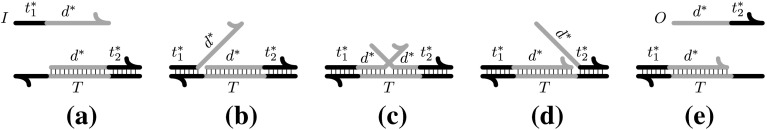
Strand displacement.** a** An unbound DNA strand *I*, with a short toehold (*dark line*) and long-domain (*lighter line*), plus a duplex consisting of a template strand *T* and a third strand *O* that is bound to *T*.** b** *I* binds to *T* via its toehold.** c** Through a process of branch migration, the long-domain of *I* becomes bound to *T*, displacing bonds of *O*.** d** *O* is bound to *T* by only a toehold.** e** The toehold bonds break, making *O* unbound

 The second main contribution of this paper is a limit on the types of DSDs that are correct in multi-copy settings. In (toehold-mediated) strand displacement (Fig. 2), an initially unbound “signal” strand *I* binds to a “template” *T*, causing another signal strand *O* that was initially bound to *T* to become unbound. DSDs are collections of strands that can change configurations via successive strand displacements in a pre-programmed fashion (Cardelli 2010; Zhang and Seelig 2011; Zhang et al. 2007) we provide a formal definition later. We do not allow other types of strand displacements, such as cooperative strand displacements, where two signal strands are needed to displace one signal strand. We thus refer to the DSDs in this paper as Uncooperative DSDs (UDSDs).

**Fig. 3:**
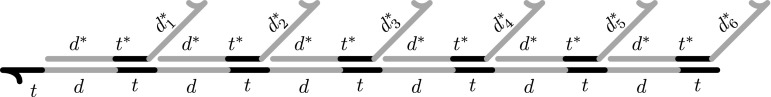
A template indicated by the* bottom line* has 6 long-domains and 6! = 720 possible configurations. The signals are the bent* top lines*.* Dark lines* are toeholds and the* lighter ones* are long-domains. The template contains δ = 6 toehold-long-domain-toehold blocks. In each block, any one of the signal species shown may be bound. Thus the number of possible configurations of this template is δ! = 6!. If 6 copies of the signal t*d* are present as well, they can displace 6 signals shown, which can subsequently displace six t*d* signals resulting in any of the 6! configurations

Our first result on tagged CRNs implies a reachability upper bound for DSD realizations of CRNs, but says nothing about DSDs more generally. In Theorem 8 we elucidate this simple upper bound which is obtained by applying the CRN result to limited types of DSDs, those whose signal strands consist of exactly two domains: a toehold and a long-domain. However, since the signal types are limited, this result does not apply to general DSDs. This is because, while tagged CRNs can be translated to DSDs having parameters such as the volume and the number of types of reactants polynomial in the volume of the CRN (Soloveichik et al. 2011), it is not clear whether the converse is true. To see why, consider signal strands that have three domains: a toehold and two long-domains such that they each start with the same long-domain d* and toehold t*, and end with a distinct long-domain. Assume there are δ different types of these signal strands where δ is the number of long-domains on the template we will consider. Note for the DSD template having δ long-domains, over the course of several displacements, there are factorially many different configurations—ways in which signal strands are bound to the template. Figure 3 provides a simple example where any permutation of the signal species could bind to the template. Now, we want to create a tagged CRN that is equivalent to this DSD. Such a tagged CRN in which each template configuration is a distinct species would thus have the number of distinct species and reactions factorial in the volume (number of toeholds and long-domains) of the DSD. Since each reaction in the tagged CRN requires a unique tag which needs to be present in the initial configuration, the overall volume of the tagged CRN would be also factorial in the volume of the DSD. It is not clear how else to translate such a DSD to a (tagged) CRN of comparable volume.

Can “long” computations be correctly performed by DSDs, even in the presence of many copies? Our second reachability upper bounds for UDSDs, Theorems 9 and 10, answer this in the negative, showing that, if sufficiently many copies are present, then any unbound DNA strand that can be produced (i.e., reached) by a sequence of strand displacements can always be reached within a number of displacements that grows at most polynomially in the volume of the single-copy UDSD. Thus, for example, we cannot write DSD programs that run correctly in the multi-copy setting and for which the minimum number of displacements needed to produce some given signal strand is exponential in the initial volume.

**Fig. 4:**
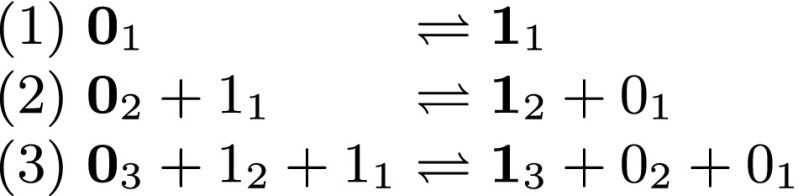
Binary counter CRN

As further motivation, we describe another application of our DSD reachability bound. The CRN of Fig. 4 describes a traditional 3-bit binary counter. Initially, three species, namely 0_3_, 0_2_ and 0_1_ represent the bits 0 at each index of the counter. Exactly one reaction can advance the counter from each value (all in the forward direction), until the counter reaches 1_3_ 1_2_ 1_1_. For the *n*-bit generalization of this counter, the number of species is just 2*n* (two species per bit) while the number of steps is 2^*n*^. Thus the volume is logarithmic in the number of steps. Another very nice feature of this CRN is that it works correctly even if multiple copies of the initial species are present, not only in the sense of being copy-tolerant but also in the sense that the trace of the multi-copy system is an interleaving of traces of the single-copy system, even in the presence of cross-talk. This follows from the fact that for every *i*, to produce each copy of molecule 1_*i*_, reactions (*i*) and (*i* − 1) have to be executed at least once in forward direction, (*i* − 2) at least twice, …, (1) at least 2^*i*−2^ times, which can be proved by induction.

However, if tags are added to the counter in order that it can be translated to a DSD using tags as discussed previously, the volume of species for the DSD realization of the counter becomes exponential in *n*. This is because reaction (1) is executed in the forward direction 2^*n*−1^ times and is never executed in the reverse direction; thus 2^*n*−1^ copies of the tag on the left side of reaction (1) must be present initially. Is there an alternative (tag-less) DSD realization of the *n*-bit CRN binary counter whose volume grows polynomially in *n*? Our DSD result implies that there is no such realization. If there were, then our reachability upper bound implies that in the multi-copy setting the bit 1_*n*_ could be produced in a polynomial number of steps. But since we know that it takes 2^*n*−1^ steps to produce 1_*n*_ even in the multi-copy setting, we have a contradiction.

## Reachability upper bound for CRNs

In this section we first provide formal definitions of tagged CRNs. We then provide our main technical result, restate this result to obtain our reachability upper bound theorem for copy-tolerant CRNs, compare the bounds of our main theorem of Sect. 2 with our previous result (Condon et al. 2012), and then provide several additional results.

### Definition of tagged CRNs

**Notation**. If $\mathcal{S}$ is a multiset, we will denote the set of distinct elements in $\mathcal{S}$ as $[\![\mathcal{S}]\!]$. If *s* is an element and *k* is a positive integer, then $k\cdot s$ denotes *k* copies of *s*. For example, a multiset containing three copies of *a* and five copies of *b*, can be represented as $\{3\cdot a,5\cdot b\}$. If *S* is a set and *k* is a positive integer, then $k\cdot S$ denotes the multiset containing *k* copies of each element in *S*. Similarly, if $\mathcal{S}$ is a multiset, then $k\cdot \mathcal{S}$ denotes the union of *k* copies of $\mathcal{S}$. The set operations on multisets are defined in a usual way. Let $\#\{x\in \mathcal{S}\}$ denote the number of copies of *x* in $\mathcal{S}$. In addition, we define the intersection $\mathcal{S}\cap T$ of a multiset $\mathcal{S}$ and a set *T* as $\mathcal{S}\cap (|\mathcal{S}|\cdot T),$ i.e., $\mathcal{S}\cap T$ contains only elements in $[\![\mathcal{S}]\!]\cap T,$ and for each $x\in [\![\mathcal{S}]\!]\cap T, \#\{x\in \mathcal{S}\cap T\} = \#\{x\in \mathcal{S}\}$.

#### **Definition 1**

(*Tagged CRN*) A tagged chemical reaction network is a tuple ${\bf C} = \langle S,T,R,\mathcal{S}_{0},\mathcal{T}_{0}\rangle$ with variables defined as follows:

- *S* is a set of signal species and *T* is the set of tag species, and *S* ∩ *T* = ∅.
- *R* is a set of reversible or irreversible reactions, where each $r\in R$ is an ordered pair $(\mathcal{I}_{r},\mathcal{P}_{r})$ of multisets of signal and tag molecules such that $\mathcal{I}_{r}\cap T = \{\tau_{r}^{+}\}$ and $\mathcal{P}_{r}\cap T = \{\tau_{r}^{-}\}$. Note that each side of each reaction contains exactly one tag molecule and this tag molecule is unique for that reaction. Intuitively, a reaction $r = (\mathcal{I}_{r},\mathcal{P}_{r})$ either consumes the molecules in $\mathcal{I}_{r}$ and produces the molecules $\mathcal{P}_{r},$ or, if the reaction is reversible, it can also consume $\mathcal{P}_{r}$ and produce $\mathcal{I}_{r}$. In the first case, we say that the reaction was applied in the *forward* direction and denote it as +*r*, in the second case in the *backward* direction and denote it as −*r*. The symbols +*r* and −*r* will be called *oriented* reactions and we define |+*r*| = |−*r*| =*r*. We will refer to $\mathcal{I}_{r}$ and $\mathcal{P}_{r}$ as the *left side* and the *right side* of a forward reaction +*r*, and as the *right side* and the *left side* of a backward reaction −*r*.
- $\mathcal{S}_{0}$ is a multiset of signal molecules and $\mathcal{T}_{0}$ is a multiset of tag molecules present initially at time-step zero. The *volume* of CRN **C** is the number of molecules in $\mathcal{S}_{0}\cup \mathcal{T}_{0}$.

Tags limit the number of times a reaction can be applied in the same direction without being applied in the reverse direction. For example, if *r* is a reversible reaction and $\mathcal{T}_{0}$ contains only one copy of* τ*_*r*_^+^ and no copies of* τ*_*r*_^−^, then in any valid trace, the oriented occurrences of *r* have to alternate, starting with +*r*. If *r* is an irreversible reaction and $\mathcal{T}_{0}$ contains *x* copies of* τ*_*r*_^+^, then in any valid trace, there are at most *x* occurrences of +*r* (and no occurrences of −*r*). Limiting the number of tags forces a system to recycle molecules in long traces.

In the following series of definitions, consider a tagged CRN system ${\bf C} = \langle S,T,R,\mathcal{S}_{0},\mathcal{T}_{0}\rangle$.

#### **Definition 2**

(*Bandwidths*) Define the bandwidth of signal species *s* as the maximum number of occurrences of *s* in $\mathcal{I}_{r}$ or $\mathcal{P}_{r},$ i.e., $\max_{r\in R} \{\#\{s\in\mathcal{I}_{r}\},\#\{s\in\mathcal{P}_{r}\}\}$. Define the maximum bandwidth *b*_**C**_ (respectively, *total bandwidth* *B*_**C**_) of **C** as the maximum (respectively, the sum) of bandwidth over all signal species in *S*. Similarly, the proper bandwidth of signal species *s*, the maximum proper bandwidth $\tilde b_{{\bf C}}$ and the total proper bandwidth $\tilde B_{{\bf C}}$ are defined analogously but using $\mathcal{I}_r \setminus  \mathcal{P}_r$ instead of $\mathcal{I}_{r}$ and $\mathcal{P}_r \setminus \mathcal{I}_r$ instead of $\mathcal{P}_{r}$.

To illustrate the above definition, consider the CRN **C** that consists of two reactions, $A+B \rightleftharpoons A+C$ and $B+B \rightleftharpoons C$. Now the respective bandwidths of the species *A*, *B*, and *C* are 1, 2, and 1, the maximum bandwidth *b*_**C**_ = 2 and the total bandwidth *B*_**C**_ = 4. Similarly, the respective proper bandwidths of the species *A*, *B*, and *C* are 0, 2, and 1, the maximum proper bandwidth $\tilde b_{{\bf C}}=2$ and the total proper bandwidth $\tilde B_{{\bf C}}=3$.

#### **Definition 3**

(*Numbers of occurrences of tags*) For any reversible reaction $r\in R,$ let *t*_*r*_ be the maximum of the number of occurrences of* τ*_*r*_^+^ or* τ*_*r*_^−^ in $\mathcal{T}_{0},$ i.e., $\max \{\#\{\tau_{r}^{+}\in  \mathcal{T}_{0}\}, \#\{\tau_{r}^{-}\in \mathcal{T}_{0}\}\};$ and for any irreversible reaction $r\in R,$ let *t*_*r*_ be the number of occurrences of* τ*_*r*_^+^ in $\mathcal{T}_{0}$. Let *T*_**C**_ be the sum of *t*_*r*_’s over all reactions $r\in R$.

#### **Definition 4**

(*x*-*copy CRN*) We define the *x*-*copy* of **C**, for ${x \in \mathbb{Z^+}, }$ as the CRN $\langle S,T,R,x\cdot \mathcal{S}_{0},x\cdot \mathcal{T}_{0}\rangle$.

#### **Definition 5**

(*Trace*) Let $\rho = r_1,r_2,\dots,r_m$ be a sequence of oriented reactions where $|r_i| \in R$ for all *i*. For oriented reaction *r* if sign(*r*) =  +, let $\mathcal{A}_r = \mathcal{I}_r$ and $\mathcal{B}_r = \mathcal{P}_r$ whereas if sign(*r*) =  −, let $\mathcal{A}_r = \mathcal{P}_r$ and $\mathcal{B}_r = \mathcal{I}_r$. The configuration of the system at each step *i* is defined as $(\mathcal{S}_i, \mathcal{T}_i)$ where $\mathcal{S}_i = (\mathcal{S}_{i-1} \setminus (\mathcal{A}_{r_{i}} \cap S)) \cup (\mathcal{B}_{r_{i}} \cap S)$ and, similarly, $\mathcal{T}_i = (\mathcal{T}_{i-1} \setminus (\mathcal{A}_{r_{i}} \cap T)) \cup (\mathcal{B}_{r_{i}} \cap T)$. A reaction sequence* ρ* is *valid* if $\mathcal{A}_{r_{i}} \cap S\subseteq \mathcal{S}_{i-1}$ and $\mathcal{A}_{r_{i}} \cap T\subseteq \mathcal{T}_{i-1}$ for all *i*, meaning that for each molecule in $\mathcal{A}_{r_{i}}$ there must be one in $\mathcal{S}_{i-1}\cup \mathcal{T}_{i - 1}$. A trace is a valid reaction sequence.

### The main upper bound

Our main upper bound, Theorem 1, shows that in the multi-copy setting, any product of a tagged CRN can be produced within a number of reactions that is bounded by a function of the number of signal species, the bandwidth, and the number of tags of the CRN.

#### **Theorem 1**

*Let* ${\bf C} = \langle S,T,R,\mathcal{S}_{0},\mathcal{T}_{0}\rangle$ *be a tagged CRN and let* $s_{{\rm end}}\in S$. *If some trace of* **C** *produces* *s*_end_, *then in a* $(|S| - |[\![\mathcal{S}_{0}]\!]| + 1)(b_{{\bf C}} + \tilde  b_{{\bf C}}T_{{\bf C}}/2)\le |S|b_{{\bf C}}(T_{{\bf C}}/2 + 1)$-*copy CRN of* **C**, *the length of the shortest trace that produces* *s*_end_ *is at most* $(|S| - |[\![\mathcal{S}_{0}]\!]|)(b_{{\bf C}} + \tilde b_{{\bf C}}T_{{\bf C}}/2)T_{{\bf C}}\le  (|S| - 1)b_{{\bf C}}(T_{{\bf C}}/2 + 1)T_{{\bf C}}$.

#### *Proof*

Let $\rho =r_1,r_2,\dots,r_m$ be a valid sequence of oriented reactions in a single-copy system producing *s*_end_ starting from the initial set $\mathcal{S}_0\cup \mathcal{T}_{0}$. We will construct a reaction sequence $\rho^{\prime}$ that also produces *s*_end_ in a multi-copy CRN and satisfies the length-bound of the theorem, by first constructing “unidirectional” shortened reaction subsequences by eliminating all forward-backward pairs of reactions {+*r*,−*r*} in subsequences of* ρ*, and then showing that in a multiple-copy setting the intermediate signals required to drive the synthesis can instead be produced by repeating these shortened reaction subsequences of* ρ*. Throughout the proof we will illustrate the construction on the CRN and the corresponding trace from Fig. 1.

Consider any prefix of this sequence, say $\rho_i=r_1,\dots,r_i$. Construct a new sequence $\rho^{\prime}_i$ by randomly pairing +*r* with −*r*, for any reaction $r\in R$, and removing these pairs from the sequence, until no such pairs can be formed, i.e., $\rho_{i}^{\prime}$ does not contain either +*r* or −*r*, for every $r\in R$. For example, consider $\rho_{6}^{\prime}$ =  +*r*_1_,+*r*_2_,−*r*_1_,+*r*_3_,+*r*_1_,+*r*_4_ from Fig. 1b. Then $\rho_{6}^{\prime} =  +r_{2},+r_{3},+r_{1},+r_{4}$ or $\rho_{6}^{\prime} =  +r_{1},+r_{2},+r_{3},+r_{4},$ depending on the choice of the +*r*_1_ and −*r*_1_ pair.

The constructed reaction sequence $\rho_{i}^{\prime}$ has the same effect on the final number of signals as* ρ*_*i*_. However, $\rho_{i}^{\prime}$ might not be a valid reaction sequence starting at the same initial configuration as* ρ*_*i*_ since some reactants might be missing when running a reaction in $\rho_{i}^{\prime}$. To avoid that we will start with a sufficient number of copies of signals in $\mathcal{S}_{0}\cup \mathcal{T}_{0}$ and run each $\rho_{i}^{\prime}$ that produces a new signal *s*_*j*_ sufficient number of times so that we have sufficient number of copies of *s*_*j*_ for all remaining executions of such shortened reaction subsequences. For example, in $\rho_{6}^{\prime} = +r_{2},+r_{3},+r_{1},+r_{4}$ the missing first reaction +*r*_1_ produces a signal *B* which is used by the subsequent reaction +*r*_2_. We can provide the missing signal *B* by running a shortened sequence $\rho_{1}^{\prime}$ that produces *B* before executing sequence $\rho_{6}^{\prime}$. In what follows we will argue that if we start in a configuration with a sufficient number of copies of signals in $\mathcal{S}_{0}  \cup \mathcal{T}_{0} \cup S_{i - 1}$, the constructed reaction sequence $\rho_{i}^{\prime}$ becomes valid.

Let *S*′ be the set of signal molecules appearing on the left hand side of reactions in $\rho^{\prime}_{i}$. Now, let us see what happens if we apply this sequence on the initial set $\mathcal{S}_0\cup \mathcal{T}_0\cup  k\cdot S'$, where *k* is sufficiently large so that the reaction sequence is valid. We can make the following observations:

1. The final number of copies of each signal species is the same as if we would apply* ρ*_*i*_ on $$\mathcal{S}_0\cup \mathcal{T}_0\cup k\cdot S'$$. Hence, the final configuration contains $$k\cdot S'$$.
2. For each reaction $$r\in R,\, \rho^{\prime}_i$$ contains either only forward or only backward occurrences of *r* (or no occurrences), and their number is limited by the number *t*_*r*_ of corresponding tags in $$\mathcal{T}_0$$. As a consequence, the length of $$\rho^{\prime}_i$$ is at most *T*_**C**_.
3. Consider a signal molecule $$s\in S'$$. Each reaction in $$\rho^{\prime}_i$$ removes or adds at most *b*_**C**_ copies of *s* and the length ℓ of $$\rho^{\prime}_i$$ is at most *T*_**C**_. We will show that before each reaction in $$\rho^{\prime}_i, $$ there are at least $$k-\tilde b_{{\bf C}}T_{{\bf C}}/2$$ copies of *s*. Assume that after the first *j* reactions, the number of copies of *s* is less than $$k-\tilde b_{{\bf C}}\ell/2$$. If $${j \leq {\ell/2}}$$, then the first *j* reactions of $$\rho^{\prime}_{i}$$ could remove at most $$\tilde b_{{\bf C}}j\le \tilde b_{{\bf C}}\ell/2$$ copies of *s*, and there were at least *k* copies present initially, a contradiction. If $${j > {\ell/2}}$$, then there are less than $${\ell/2}$$ reactions left, and each of them adds at most $$\tilde b_{{\bf C}}$$ copies of *s*. Since by (1), the final number of copies of *s* is at least *k*, we have a contradiction again. Hence, the number of copies of *s* before any reaction of $$\rho^{\prime}_{i}$$ is at least $$k-\tilde b_{{\bf C}}\ell /2\ge k-\tilde b_{{\bf C}}T_{{\bf
C}}/2$$.
4. Hence, it follows that if we set $$k = b_{{\bf C}} + \tilde b_{{\bf C}} T_{{\bf C}}/2$$, then before each reaction in $$\rho^{\prime}_i, $$ there are at least *b*_**C**_ copies of any signal in *S*′, and hence, the reaction sequence is valid. Note that this is true even if we randomly permute reactions in $$\rho^{\prime}_{i}$$.

For each signal *s* appearing in the single-copy trace and not appearing in the initial set $\mathcal{S}_{0}$, let $r_{{\rm index} (s_{i})}$ be the first reaction in* ρ* which produces a copy (or more) of *s*. Let $s_1,\ldots,s_n$ be the sequence of all signals not in $\mathcal{S}_{0}$ ordered by their indices, i.e., ${\rm index}(s_1) \le {\rm index}(s_2) \le \cdots \le {\rm index}(s_n)$. In our example from Fig. 1, we have index(*B*) = 1, index(*D*) = 2, index(*E*) = 4 and index(*F*) = 6. Hence, we order signals as follows: *s*_1_ = *B*, *s*_2_ = *D*, *s*_3_ = *E* and *s*_4_ = *F*.

Without loss of generality we can assume *s*_*n*_ = *s*_end_. Let $S_{i} = \{s_1,\dots,s_{i}\}$. We can make one additional observation:

(5) For each *s*_*i*_, the left side of each reaction in $\rho^{\prime}_{{\rm index}(s_i)}$ contains only signals in $[\![\mathcal{S}_0]\!] \cup S_{i-1}$. By (4), if we start in a configuration which contains the multiset of signals and tags $\mathcal{S}_{0}\cup \mathcal{T}_{0} \cup (b_{{\bf C}} + \tilde b_{{\bf C}}T_{{\bf C}}/2)\cdot ([\![\mathcal{S}_0]\!] \cup S_{i-1}), \rho^{\prime}_{{\rm index} (s_{i})}$ is a trace producing a copy of *s*_*i*_.

#### Construction of reaction sequence

1. Start with the initial set containing $b_{{\bf C}} + \tilde b_{{\bf C}} T_{{\bf C}}/2$ copies of $[\![\mathcal{S}_0]\!]$ and the empty sequence of reactions.
2. For each $i=1,\dots,n:$ add $b_{{\bf C}} + \tilde b_{{\bf C}} T_{{\bf C}}/2$ copies of $\mathcal{S}_0\cup \mathcal{T}_{0}$ to the initial set and append $b_{{\bf C}} + \tilde b_{{\bf C}} T_{{\bf C}}/2$ times sequence $\rho^{\prime}_{{\rm index}(s_i)}$ to the constructed sequence of reactions.

Before we proceed with proving that this construction is producing a valid reaction sequence, let us illustrate it on the CRN from Fig. 1. Since *T*_**C**_ = 4 and $b_{{\bf C}}  = \tilde b_{{\bf C}} = 1,$ we have $b_{{\bf C}} + \tilde  b_{{\bf C}}T_{{\bf C}}/2 = 3$. The construction starts by putting $\{3\cdot A,3\cdot C\}$ into the initial set and proceeds in four steps:

1. *ρ*_index(*B*)_ =  +*r*_1_, and hence, $\rho^{\prime}_{\text{index}(B)}$ =  +*r*_1_. We add $\{3\cdot A,3\cdot C\}\cup 3\cdot \mathcal{T}_{0}$ into the initial set and start constructing a new reaction sequence with +*r*_1_,+*r*_1_,+*r*_1_.
2. *ρ*_index(*D*)_ =  +*r*_1_,+*r*_2_, and hence, $\rho^{\prime}_{{\text{index}}(D)}$ =  +*r*_1_,+*r*_2_. We add $\{3\cdot A,3\cdot C\}\cup 3\cdot \mathcal{T}_{0}$ into the initial set and append +*r*_1_,+*r*_2_,+*r*_1_,+*r*_2_,+*r*_1_,+*r*_2_ to the constructed sequence.
3. *ρ*_index(*E*)_ = +*r*_1_,+*r*_2_,−*r*_1_,+*r*_3_, and hence, $\rho^{\prime}_{{\text{index}}(E)}$ = +*r*_2_,+*r*_3_. We add $\{3\cdot A,3\cdot C\}\cup 3\cdot \mathcal{T}_{0}$ into the initial set and append +*r*_2_,+*r*_3_,+*r*_2_,+*r*_3_,+*r*_2_,+*r*_3_ to the constructed sequence.
4. *ρ*_index(*F*)_ =  +*r*_1_,+*r*_2_,−*r*_1_,+*r*_3_,+*r*_1_,+*r*_4_, and we choose the second option $\rho^{\prime}_{{\text{index}}(F)}$ =  +*r*_1_,+*r*_2_,+*r*_3_,+*r*_4_. We add $\{3\cdot A,3\cdot C\}\cup 3\cdot \mathcal{T}_{0}$ into the initial set and append +*r*_1_,+*r*_2_,+*r*_3_,+*r*_4_,+*r*_1_,+*r*_2_,+*r*_3_,+*r*_4_,+*r*_1_,+*r*_2_,+*r*_3_,+*r*_4_, to the constructed sequence.

**Fig. 5:**
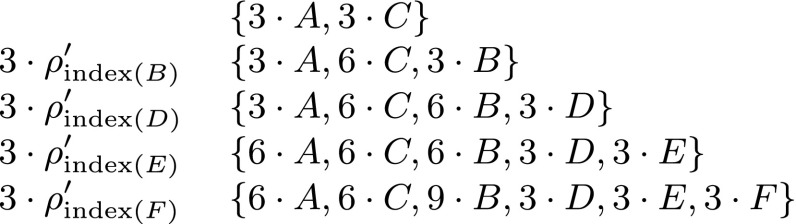
The intermediate configurations after each step of the construction applied to the CRN in Fig. 1a. The multisets in the second column show only the signal species

Hence, the construction requires 15 copies of $\mathcal{S}_{0}$ and 12 copies of $\mathcal{T}_{0},$ and the constructed reaction sequence contains 27 reactions. This is not the most efficient sequence. As we have seen in the introduction, there is a reaction sequence of length four that uses only two copies of $\mathcal{S}_{0}$ and one copy of $\mathcal{T}_{0},$ and produces a signal species *F*. However, this general construction guarantees that the required number of copies of the initial set and the length of the sequence is polynomial for any CRN. The configuration of the system after each step is shown in Fig. 5. Note that after each step the configuration contains at least three copies of each species produced so far. We will show that this is always the case in the following claim, which also proves that the constructed sequence is valid.

##### **Claim 1**

*After each step* *i in (S2), the constructed sequence is valid and the final configuration contains* $b_{{\bf C}} + \tilde b_{{\bf C}} T_{{\bf C}}/2$ *copies of each signal in* $[\![\mathcal{S}_0]\!] \cup S_i$.

##### *Proof*

Proof by induction: *Base case:* For *i* = 0, after (S1), we have $b_{{\bf C}} + \tilde b_{{\bf C}} T_{{\bf C}}/2$ copies of each signal in $[\![\mathcal{S}_0]\!]$ and the empty sequence of reactions is valid. *Induction step:* Inductive assumption: before step *i*, we have $b_{{\bf C}} + \tilde b_{{\bf C}} T_{{\bf C}}/2$ copies of each signal in $[\![\mathcal{S}_0]\!] \cup S_{i-1}$ and the sequence constructed so far is valid. By (5), if we add a copy of $\mathcal{S}_0\cup \mathcal{T}_{0}$ and apply the reaction sequence $\rho^{\prime}_{{\rm index}(s_i)}$ on the current configuration, the trace is valid. By (1), this newly added part (a copy of $\mathcal{S}_{0}\cup \mathcal{T}_{0}$ and reactions in $\rho^{\prime}_{{\rm index}(s_i)}$) will not decrease the number of any signal. Finally, $\rho^{\prime}_{{\rm index}(s_i)}$ must contain the last reaction of* ρ*_index_(*s*_*i*_), i.e., *r*_index_(*s*_*i*_) which produces at least one copy of *s*_*i*_. If we repeat this $b_{{\bf C}} + \tilde b_{{\bf C}} T_{{\bf C}}/2$ times, we will still have at least $b_{{\bf C}} + \tilde b_{{\bf C}} T_{{\bf C}}/2$ copies of signals in $[\![\mathcal{S}_0]\!] \cup S_{i-1}$ plus $b_{{\bf C}} + \tilde b_{{\bf C}} T_{{\bf C}}/2$ copies of *s*_*i*_. $\square$

The bound: The construction uses $(n+1)(b_{{\bf C}} + \tilde b_{{\bf C}} T_{{\bf C}}/2)$ copies of $\mathcal{S}_0,$ $n(b_{{\bf C}} + \tilde  b_{{\bf C}} T_{{\bf C}}/2)$ copies of $\mathcal{T}_0$ and repeats $n(b_{{\bf C}} + \tilde  b_{{\bf C}} T_{{\bf C}}/2)$ times the trace $\rho^{\prime}_{{\rm some\, index}}$. By (2), the length of each $\rho^{\prime}_{{\rm some\, index}}$ trace is at most *T*_**C**_, hence the total length of the constructed sequence is at most $n(b_{{\bf C}} + \tilde b_{{\bf C}} T_{{\bf C}}/2)T_{{\bf C}}$. Furthermore, *n* can be bounded by $|S| - |[\![\mathcal{S}_{0}]\!]|$. $\square$

We remark that this result does not hold for the untagged CRNs, cf. Example 3.4 in our earlier paper (Condon et al. 2012), where the following CRN was presented which requires an exponential number of steps to produce *s*_end_ even in an $\infty$-copy of this CRN:

$$ s_{i} + s_{i}\rightleftharpoons s_{i + 1} + s_{0},\quad \text{for} \quad i = 0,\dots,k-1, $$

with the initial set containing *k* copies of *s*_0_. Note that since all reactions are balanced the volume of this system stays constant. However, since in this CRN in any shortest trace producing *s*_end_, all reactions are applied in the forward direction, if we would tag this CRN, the trace producing *s*_end_ would require that the initial multiset of tag molecules $\mathcal{T}_{0}$ contains an exponential number of tags. It is also interesting to observe where the proof of Theorem 1 fails for the untagged CRNs. In observation (2) in the proof we were able to bound the length of the shortened sequence of reactions $\rho_{i}^{\prime}$ by *T*_**C**_, which would not be possible in an untagged CRN.

Next, we restate Theorem 1 for copy-tolerant CRNs.

##### **Theorem 2**

*If a tagged CRN* $${\bf C} = \langle S,T,R,\mathcal{S}_{0},\mathcal{T}_{0}\rangle$$ *is* $${|{S}|{b_{C}}\!\left({T_{C}}/2 + 1\right)}$$-*copy-tolerant and* *s*_end_ *can be produced in* **C**, *then the length of the shortest trace of* **C** *that produces* *s*_end_ *is at most* $${\left(|{S}|-1\right)\!{b_{C}}\!{\left({T_{C}}/2 +
1\right)\!{T_{C}}}}$$.

A natural question is whether we could improve the bound in condition (3) of the proof of Theorem 1 by choosing the “right” permutation of oriented reactions in $\rho_{i}^{\prime}$. The following example shows that this is not possible in general.

##### *Example 1*

Assume that* ρ* contains exactly an even number, *T*, of oriented reactions $+r_{1},\dots,+r_{T}$ designed as follows. First for every partition π of* ρ* into two sets *ρ*_1_^π^ and* ρ*_2_^π^ of same size, we introduce a new signal *s*_π_. Let $\Uppi$ be the set of all such partitions. Next, we define reactions $r_{1},\dots,r_{T}$ in such a way that each of these signals is either an input or a product of each reaction:

$$ \begin{aligned} {\mathcal{I}}_{r_{i}} &= \{s_{\pi }:\ r_{i}\in \rho_{1}^{\pi },\pi \in \Uppi \} ,\\ {\mathcal{P}}_{r_{i}} &= \{s_{\pi }:\ r_{i}\in \rho_{2}^{\pi },\pi \in \Uppi \} \end{aligned} $$

We will illustrate this construction for *T* = 4, i.e.,* ρ* =  +*r*_1_,+*r*_2_,+*r*_3_,+*r*_4_. There are six partitions of* ρ* into two subsets of size two:

- *ρ*_1_^α^ =  +*r*_1_,+*r*_2_ and* ρ*_2_^α^ =  +*r*_3_,+*r*_4_, 
- *ρ*_1_^β^ =  +*r*_1_,+*r*_3_ and* ρ*_2_^β^ =  +*r*_2_,+*r*_4_, 
- *ρ*_1_^γ^ =  +*r*_1_,+*r*_4_ and* ρ*_2_^γ^ =  +*r*_2_,+*r*_3_, 
- *ρ*_1_^δ^ =  +*r*_2_,+*r*_3_ and* ρ*_2_^δ^ =  +*r*_1_,+*r*_4_, 
- *ρ*_1_^ε^ =  +*r*_2_,+*r*_4_ and* ρ*_2_^ε^ =  +*r*_1_,+*r*_3_, 
- *ρ*_1_^ζ^ =  +*r*_3_,+*r*_4_ and* ρ*_2_^ζ^ =  +*r*_1_,+*r*_2_, 

Hence, the reactions use six signals: $s_{\alpha },s_{\beta },s_{\gamma },s_{\delta },s_{\epsilon },s_{\zeta }$. Using the definition of inputs and products above, the four constructed reactions are the following:

$$ \begin{array}{llll} r_{1}:\quad {s_{\alpha } + s_{\beta } + s_{\gamma }} & \rightleftharpoons \quad {s_{\delta } + s_{\epsilon } + s_{\zeta }} \\ r_{2}:\quad {s_{\alpha } + s_{\delta } + s_{\epsilon }} & \rightleftharpoons \quad {s_{\beta } + s_{\gamma } + s_{\zeta }} \\ r_{3}: \quad {s_{\beta } + s_{\delta } + s_{\zeta }} & \rightleftharpoons \quad {s_{\alpha } + s_{\gamma } + s_{\epsilon }} \\ r_{4}:\quad{s_{\gamma } + s_{\epsilon } + s_{\zeta }} & \rightleftharpoons \quad {s_{\alpha } + s_{\beta } + s_{\delta }} \\ \end{array} $$

Note that after all reactions in* ρ* are applied, the number of copies of any of the signals *s*_π_ is not changed, since there are exactly ${{T}/2}$ reactions in* ρ* adding one copy of *s*_π_ and ${{T}/2}$ reactions removing one copy of *s*_π_.

Now, we show that for any permutation of the reactions in* ρ*, there is a signal molecule with *k* − ${{T}/2}$ copies when the first ${{T}/2}$ reactions in this order are applied. Since we could easily replace $\mathcal{I}_{r_{i}}$ and $\mathcal{P}_{r_{i}}$ with $b\cdot \mathcal{I}_{r_{i}}$ and $b\cdot \mathcal{P}_{r_{i}},$ the bound $k - \tilde b_{{\bf C}}T/2$ in (3) cannot be improved without adding some additional conditions on the CRN. To find the signal molecule with *k* − ${{T}/2}$ copies after applying the first ${{T}/2}$ reactions, consider the partition π_0_ of* ρ* into the first and the second ${{T}/2}$ reactions of this order. Then the signal $s_{\pi_0}$ appears in the input set of the first ${{T}/2}$ reactions, and thus, the number of copies of $s_{\pi_0}$ is *k* − ${{T}/2}$ after applying the first ${{T}/2}$ reactions.

#### Result for 1-proper tagged CRNs

We next describe a stronger version of our result for a special case. We say that a tagged CRN **C** is *k*-*proper* if each reaction has at most *k* reactants which are not catalysts, more formally, for all $r \in R,$ $|\mathcal{I}_r \setminus \{\tau_r^+\} \setminus \mathcal{P}_r| \le k$ and if *r* is reversible, also $|\mathcal{P}_r \setminus \{\tau_r^-\}\setminus \mathcal{I}_r| \le k$.

##### **Corollary 1**

*If there exists a trace in a 1-proper tagged CRN* ${\bf C} = \langle S,T,R,\mathcal{S}_{0},\mathcal{T}_{0}\rangle$ *producing* *s*_end_, *then in an* |*S*|*b*_**C**_-*copy CRN of* **C**, *the length of the shortest trace that produces* *s*_end_ *is at most* (|*S*| − 1)*b*_**C**_ *T*_**C**_.

##### *Proof*

To improve the bound we will strengthen the bound for *k* in observations (1–4) in the proof of Theorem 1. In particular, we will show that there is a permutation of reactions in $\rho^{\prime}_{i}$ such that when this permutation of reactions is applied on $\mathcal{S}_{0}\cup \mathcal{T}_{0}\cup b_{{\bf C}}\cdot S'$, the number of copies of any signal species is not below *b*_**C**_ − 1 during any step and the number of copies of any but one signal is not below *b*_**C**_. To do this we will borrow the idea from the proof of Theorem 2 in (Condon et al. 2012). Pick the first reaction at random. Since it is a 1-proper reaction, the number of copies of at most one signal species, say *s*, is less than *b*_**C**_, and if so, by at most one less. By (1), there has to be an unused reaction which would bring this number back to *b*_**C**_. We choose this reaction as the second reaction. This brings the number of copies of molecule species *s* back to *b*_**C**_, but it might decrease the number copies of another species to *b*_**C**_ − 1. Hence, there is again at most one signal species with fewer than *b*_**C**_ copies. Repeating this process, we construct the desired permutation of reactions of $\rho^{\prime}_{i}$.

Using this improved bound of condition (3), we can now modify the construction of the reaction sequence as follows:

1. Start with *b*_**C**_ copies of $[\![\mathcal{S}_0]\!]$.
2. For each $i=1,\dots,n$: add *b*_**C**_ copies of $\mathcal{S}_0\cup \mathcal{T}_{0}$ and append *b*_**C**_ times sequence $\rho^{\prime}_{{\rm index}(s_i)}$.

The rest of the proof follows analogously to the proof of Theorem 1.$\square$

#### Comparison with the previous result

In our previous work (Condon et al. 2012), we have showed the following result for untagged CRNs. Untagged CRNs do not put any restriction on how many times reactions are used in forward or backward directions. They can be also thought of as tagged CRNs with an infinite supply of tags [for the exact definition see (Condon et al. 2012)].

##### **Theorem 6**

(Condon et al. 2012). *If there exists a trace in a 1-proper CRN* ${\bf C} = \langle  S,R,\mathcal{S}_{0}\rangle$ *producing* *s*_end_, *then in a* (*B*_**C**_ + 1)-*copy CRN of* **C**, *the length of the shortest trace that produces* *s*_end_ *is at most* (*B*_**C**_ + 1)*B*_**C**_/2 + 1.

Note that *B*_**C**_ ≤ |*S*|*b*_**C**_. In particular, if the maximum bandwidth is 1, then the number of copies of the system required in both results is $\Uptheta (|S|)$ and the number of reactions needed to produce *s*_end_ is bounded by *O*(|*S*|*T*_**C**_) in our new result and by *O*(|*S*|^2^) in the result from (Condon et al. 2012).

##### The upper bound in the unrestricted case

 In the previous subsection, we assumed that a single copy CRN can produce the target signal molecule *s*_end_. Here we study the case without this assumption. We have the following weaker result:

###### **Theorem 7**

*Consider a CRN* ${\bf C} = \langle S,R,\mathcal{S}_{0}\rangle$ *with the maximum bandwidth* 1. *If* *s*_end_ *can be produced by an* $\infty$-*copy CRN of* **C**, *then the length of the shortest trace that produces* *s*_end_ *is at most* *O*(2^|*R*|^) *in the* $\infty$-*copy CRN of* **C**.

###### *Proof*

Partition *R* as follows. In an $\infty$-copy CRN, we can assume that we have an unlimited supply of signal molecules in $\mathcal{S}_{0}$. Let *R*_1_ be the set of all reactions in *R* which can be applied in the initial configuration. Let *S*_1_ be the set of signal molecules in $\mathcal{S}_{0}$ and those produced by reactions in *R*_1_. Repeat this procedure until *S*_*k*_ contains *s*_end_. Let *r*_*i*_ be the size of *R*_*i*_. We want to estimate how many reaction steps are needed until we can apply the reaction in *R*_*k*_ that produces *s*_end_. In the worst case, to apply any reaction in *R*_*i*_, we might need signal molecules produced by each reaction in $R_{1}\cup \dots\cup R_{i-1}$. Let *b*_*i*_ be an upper bound on the number of reaction steps which will produce all signal molecules required to apply a reaction in *R*_*i*_. Hence, we can set *b*_1_ = 0 and *b*_*i*_ = ∑_*j*=1_^*i*−1^ *r*_*j*_(*b*_*j*_ + 1). Note that *b*_*i*+1_ − *b*_*i*_ = *r*_*i*_(*b*_*i*_ + 1), and hence, *b*_*i*+1_ + 1 = *r*_*i*_(*b*_*i*_ + 1) + *b*_*i*_ + 1 = (*b*_*i*_ + 1)(*r*_*i*_ + 1). And thus, $b_{i} = \prod_{j = 1}^{i - 1} (r_{j} +  1) - 1$.

To upper bound *b*_*k*_ we will argue that the value of *b*_*k*_ is maximized if *r*_*j*_ = 1 for each $j = 1,\dots,k$, and *k* = |*R*|. This is because for any *n*_1_,*n*_2_ ≥ 1 such that *n*_1_ + *n*_2_ = *n*, it holds that *n* + 1 < (*n*_1_ + 1)(*n*_2_ + 1), i.e., the product could always be increased by replacing the term *r*_*j*_ + 1 ≥ 3 in the product with two terms *r*′_*j*_ + 1 and *r*″_*j*_ + 1, where *r*_*j*_ = *r*′_*j*_ + *r*″_*j*_. The claim follows by induction. Therefore, we can upper bound the number of reactions needed to produce *s*_end_ by $b_{k} + 1 = \prod_{j = 1}^{k - 1} (r_{j} + 1)\le 2^{|R| - 1}$.$\square$

The following example shows that the bound in Theorem 7 cannot be improved.

###### *Example 2*

Consider the following CRN with the maximum bandwidth 1:

$$ s_{0} + s_{1} + \cdots + s_{i} \rightleftharpoons s_{i + 1},\quad \text{for} \quad i = 0,\ldots,k-1. $$

This CRN contains *k* distinct reactions and the number of reaction steps required to produce *s*_*k*_ is 2^*k*−1^, which exactly matches the bound in Theorem 7. Indeed, if we denote the number of reactions needed to produce *s*_*i*_ by *n*_*i*_, then we have *n*_0_ = 0 and $n_{i} = n_{0} + n_{1} + \cdots + n_{i -1} + 1$, and it is easy to check that *n*_*i*_ = 2^*i*−1^, for every *i* ≥ 1.

## Reachability upper bound for uncooperative DSDs

In this section we first define the type of DSD to which our results apply, along with related notation needed for our results. We then provide our main upper bound, and conclude with a restatement of this result to obtain our reachability upper bound theorem for copy-tolerant DSDs.

### Definition of uncooperative DSDs

In this section we formalize standard features of DSDs as described in the literature and some additional features, so that we can reason rigorously about them in our proofs. Since our model does not allow cooperative strand displacement, we call this model “uncooperative DSD” model (UDSD).

In our definition of UDSDs, we will assume that the basic building blocks are domains where each domain *d* has its complementary domain d* and (d*)* = *d*. In practice, domains are built from nucleotide sequences, and it is usually assumed that these are designed in a way so that there are no interactions between domains which are not complementary. In addition, domains are usually divided into two groups, “toeholds” and “long-domains”, based on their lengths (the number of nucleotides). Strands are built by concatenating these basic building blocks. The purpose of toeholds is to initiate branch migration [replacement of one strand (signal) attached to a long strand (template) by another strand (signal)]. The purpose of long-domains is exactly the opposite: to prevent signal strands from detaching from the template strands without being replaced by another signal strand (as this would require a prohibitive amount of energy). Consistent with existing research, we are working at the domain level of abstraction, and we consider the actual sequence design of domains as future work.

An *Uncooperative DNA strand displacement system (UDSD)* is a pair $\Updelta = ({\cal S},{\cal C}_{init})$ of strands and an initial configuration (secondary structure) for those strands, plus allowable *positional displacements*, defined as follows.

- ${\cal S}$ is a finite multiset of *strands*. Strands are composed of subsequences of finite strings of symbols, called *domains*. Domains are partitioned into two groups: *toeholds* and *long-domains*. Corresponding to each domain *x* is a complementary domain x*; *x* is a toehold if and only if x* is. ${\cal S}$ may contain many strands of a given type, where the type of a strand is its sequence of domains. The strands are partitioned into two groups: *signals* and *templates*. A template strand is a sequence of domains beginning and ending with a toehold that alternates between toeholds and long-domains. A signal strand is an arbitrary sequence of domains. There is no bound on the number of toeholds and long-domains of a template or a signal.
  We say that the UDSD $\Updelta$ has *simple signals*, if each signal in $\mathcal{S}$ is composed of exactly one toehold and one long-domain.

- A *configuration* of $\mathcal{S}$ is a circular graph 2 with the vertex set containing all domains in $\mathcal{S}$ and the edge set consisting of two types of edges: (i) *adjacency edges* connecting all adjacent domains in the strands of $\mathcal{S}$ and (ii) *binding edges* connecting some complementary domains, which satisfy the following conditions:
  1. Every domain is incident to at most one binding edge. A domain incident to a binding edge, is called *bound*, otherwise, it is called *unbound*.
  2. There are no binding edges between domains on template strands.
  3. There are no binding edges between domains on signal strands.
  4. For each template strand, all domains but one toehold domain are bound. This one unbound toehold is called the *open toehold* of the template.
  5. For every signal strand, either all its domains are unbound, in which case we say that the signal strand is *unbound*, or exactly two of its domains which are adjacent are bound to two adjacent domains on one template strand, in which case we say that the signal strand is *bound*.

In addition, since we assume that a domain is a toehold if and only if the complementary domain is a toehold, and all binding edges are between complementary domains, there are no binding edges between toeholds and long-domains. We will call the connected components of this graph *complexes*. Note that conditions (2), (3) and (5) imply that the configuration is a circular graph, hence we could have omitted it from the above definition. However, we choose to include it so that omitting condition (5) from the above definition yields a valid more general model of DSDs, which we would like to consider in future work.

For example, Fig. 7 shows two configurations, the initial (top) and final (bottom) configurations of a UDSD. This are drawn, not as a typical circular graph, but with domains (vertices) represented by lines (short dark lines represent toeholds and long gray lines represent long-domains), adjacency edges by connected domains and binding edges indicated by complementary domains that are juxtaposed. Most of the signals in these configurations are simple, and there is one complex signal that can bind to two different positions of the first template. The PDs are lined up horizontally with their template positions and given by circles that are numbered according to the order in which they occur.

**Fig. 6:**
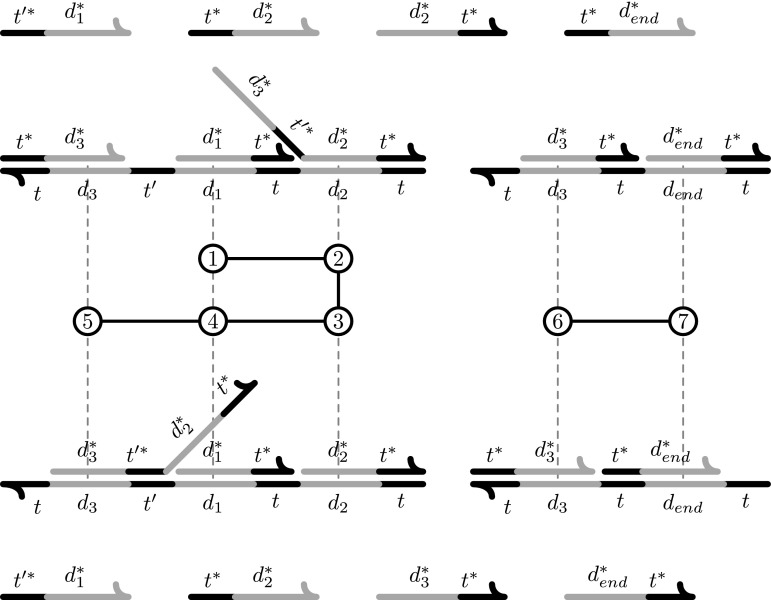
This figure shows the initial configuration *C*_*init*_ (*top*) and the final configuration (*bottom*) of a small UDSD. The UDSD has two templates and nine signals with one of them being a complex signal. The final configuration is characterized by the release of the signal *s*_*end*_ = *d*_end_^*^ t*. The middle portion of the figure illustrates the sequence of PDs that moves the UDSD from *C*_*init*_ to the release of *s*_*end*_. The PDs are ordered according to the sequence in which they occur, and the number for the PD is located horizontally over/under the template domain that is affected by the PD. The complex signal is active in two PDs, 2 and 5. PDs 1–5 interact with the first template by walking first to the right and then to the left along the template domains. This behavior is discussed in detail in Fig. 8. A more detailed illustration of the signals that participate in each PD is shown in Fig. 7

**Fig. 7:**
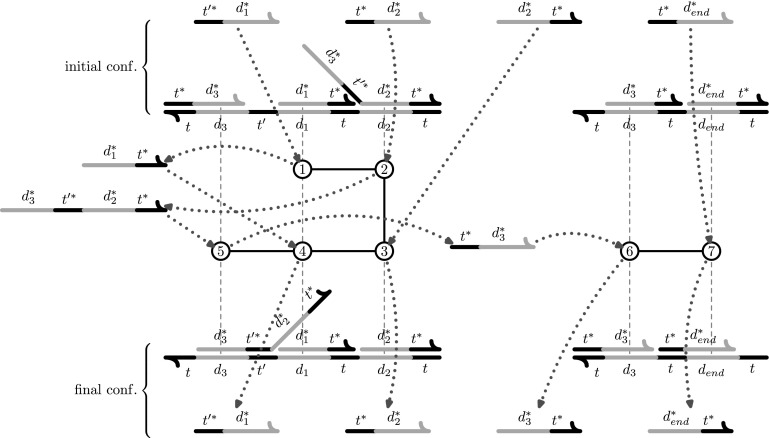
This figure shows more detail for the sequence of PDs outlined in Fig. 6. The initial configuration *C*_*init*_ is shown at the* top* and the final configuration with the release of signal *s*_*end*_ = *d*_end_^*^ t* is shown at the* bottom*. Each PD is indicated by a number in a* circle* where the numbers give the order in which the PDs occur. Each PDs* circle* has an incoming edge and an outgoing edge. The incoming edge indicates which signal is the invader and the outgoing edge indicates which signal is the release

Let us now provide some intuition behind these conditions. Condition (1) comes from the fact that each nucleotide can form a (hydrogen) bond with only one another nucleotide, and thus the same applies to domains which are sequences of nucleotides. Conditions (2) and (3) are typical assumptions made for the systems which divide strands into templates and signals (as we do). The advantage of such a design is better control of what can and cannot happen in the system. If the UDSD is designed in such a way that no domains in the signals are complementary to each other and similarly, no domains in templates are complementary to each other, then these two conditions are implied and could be dropped from the definition of the configuration. Note that these two conditions imply that the subgraph containing only binding edges is a bipartite graph.

Conditions (4) and (5) are two additional assumptions which we make to prove our results. It is possible that our results hold even if any of these two conditions or both of them are dropped. We leave that as an open problem. Condition (4) guarantees that each configuration is at the minimum free energy. Condition (5) limits how signal strands and template strands interact. If the UDSD is designed in such a way that for each signal and each template there is no scattered substring of the signal of length more than two which is complementary to a substring of the template, then the part of condition (5), which states that a signal binds to a template with exactly two adjacent domains, is implied. For example, consider a signal a*b*c*d*e* and a template *uvabdexy*. Then a scattered substring a*b*d*e* of the signal of length four could bind to a substring *abde* of the template, thus breaking condition (5). The second part of condition (5), which states that a signal strand does not simultaneously bind to two different templates, is commonly assumed in any system which we have seen in the literature and is necessary for our proofs to work.

As a consequence of these conditions we have that the only way one configuration can be transformed to another configuration is through “positional strand displacement” described below, which for example, does not allow cooperative displacement, thus the name “uncooperative DSD” for our model. (As we will see later, a sequence of strand displacements can “walk” back and forth in templates, with each displacement using toeholds that become open as a result of the previous displacement in the sequence, but such walks are necessarily restricted to remain within a template. For example, see Fig. 6.)

- ${\cal C}_{init}$ is an initial configuration.

Starting with the initial configuration, DSDs can progress through a sequence of configurations via positional strand displacements (PDs). PDs can move the open toehold of the template to the right or to the left. A PD moving the open toehold to the right is specified by a positive even number *k*, a template strand *T* with at least *k* + 1 domains and a signal strand called the *invader*, say of type *I*, see Fig. 2a, where we can now assume that only positions *k* − 1, *k*, *k* + 1 of template *T* are shown. The domain *d* at position *k* of the template is a long-domain and the domain at position *k* − 1 is a toehold, say *t*. For the displacement to be applicable to a given configuration ${\cal C},$ it must be that in ${\cal C}$ an additional signal strand, which we refer to as the *releasee*, is bound to *d* at position *k* and to a toehold at position *k* + 1 of the template *T*, and the toehold at position *k* − 1 is unbound (open). The invader is unbound in ${\cal C}$ and contains the substring t*d*.

A displacement models the following steps in Fig. 2b,c,d, when toeholds and long-domains are actual DNA sequences. First, toehold t* of the invader binds to the toehold *t* of the template at position *k* − 1. Then a branch migration ensues, whereby long-domain d* of the invader binds to *d* at position *k* of the template and the releasee is no longer bound at this position. Finally, if it exists, the bond between the releasee and the toehold at position *k* + 1 is broken. Thus in the resulting configuration ${\cal C}',$ substring t*d* of the invader is bound to *td* on the template at positions *k* − 1 and *k* and the releasee is unbound, see Fig. 2e.

Formally a positional displacement (PD) of UDSD $\Updelta$ is a tuple of the form (*I*, *T*, *k*, *z*), where *I* is a signal strand type, *T* is a template strand, *k* is a positive even integer and $z \in \{L,R\}$. PD (*I*, *T*, *k*, *z*) is *applicable* to a configuration ${\cal C}$ if the following conditions hold:

1. Strand *T* has at least *k* + 1 domains and the *k*th domain, say *d*, is a long-domain. Also a signal strand, called the releasee, is bound to the *k*th domain of *T*.
2. In the configuration ${\cal C},$ a strand of type *I* is unbound.
3. If *z* = *R* the following conditions hold. (Conditions for *z* = *L* are symmetric, with *k* + 1 swapped with *k* − 1 and d*t* replacing t*d*.)
  - The (*k* − 1)st domain of *T* must exist and is a toehold, say *t*.
  - A strand of type *I* contains substring t*d*.
  - The releasee is also bound to a toehold at position *k* + 1 of *T*. No other domains of the releasee are bound.
  - The toehold at position *k* − 1 of strand *T* is unbound. We call this toehold the *input toehold* of PD (*I*, *T*, *k*, *z*).

The PD must *release* exactly one signal strand. Suppose that PD (*I*, *T*, *k*, *z*) is applicable to ${\cal C}$. Let ${\cal C}'$ be obtained from ${\cal C}$ by removing the bonds between *T* and the releasee and by adding bonds either between any substring t*d* of an unbound strand of type *I* of ${\cal C}$ and the domains *td* at positions *k* − 1 and *k* of *T* if *z* = *R*, or between any substring d*t* of *I* and the substring *dt* at positions *k* and *k* + 1 of *T* if *z* = *L*. Then we say that (*I*, *T*, *k*, *z*) induces ${\cal C}'$ from ${\cal C}$. This definition excludes *cooperativity* where two invading strands release a single releasee or one invading strand releases two releasees, because, by definition, every PD must be initiated by one invader and release exactly one releasee.

A sequence of PDs $\rho = p_1, p_2,\ldots, p_{|\rho |}$ is *valid* with respect to ${\cal C}_{init}$ if there is a sequence ${\cal C}_1, {\cal C}_2, \ldots, {\cal C}_{|\rho |+1}$ of configurations of $\Updelta$ with ${\cal C}_1 = {\cal C}_{init}$ such that for all *i*, 1 ≤ *i* ≤ ${|\rho |}$, *p*_*i*_ is applicable to ${\cal C}_i$ and induces ${\cal C}_{i+1}$ from ${\cal C}_i$. When ${\cal C}_{init}$ is clear from the context, we simply say that* ρ* is valid. A valid sequence *produces* a strand $s \in {\cal S}$ if in ${\cal C}_{|\rho |+1},$ the strand *s* is unbound. Let Invaders(*ρ*) be the set of types of invaders of* ρ*. Let ${\rm Unbound}(\rho , {\cal C}_{init})$ be the set of types of unbound signals in ${\cal C}_{|\rho |+1}$ and Unbound(*ρ*) the set of types of unbound signals in ${\cal C}_{1}\cup\dots \cup {\cal C}_{|\rho | + 1}$. For example, Fig. 6 shows an initial configuration (top) and a final configuration (bottom) for a PD. There are two templates and nine signals, one of which is a complex signal. Each configuration shows the signals bound to the templates and the unbound signals above them.

Let $\rho = p_1, p_2,\dots,p_{|\rho |}$ be a sequence of PDs. The *template subsequence* *ρ*(*T*) is the subsequence of* ρ* with PDs of the form *p*_*i*_ = (*I*_*i*_, *T*, *k*_*i*_, *z*_*i*_) where *u* < *k*_*i*_ < *v*.

The *volume* of UDSD $\Updelta$ is the number of domains in $\mathcal{S}$.

### The upper bounds

First, we use the fact that a UDSD with simple signals can be simulated by a tagged CRN with volume that is polynomial in the volume of the UDSD, and thus we can use the bound in Theorem 1 to obtain the following result. If $\Updelta = ({\cal S},{\cal C}_{init})$ is a UDSD, we define $\Updelta^{(x)}$ to be the UDSD $(x\cdot {\cal S},x\cdot{\cal C}_{init}),$ where $x\cdot \mathcal{C}_{init}$ denotes the configuration that contains *x* copies of each complex in $\mathcal{C}_{init}$.

#### **Theorem 8**

*Let* $\Updelta$ *be a UDSD with simple signals*. *Let* *B* *be the number of types of initially bound signal strands and* *D* *be the total number of long-domains of all templates*. *If* $\Updelta$ *can produce* *s*_*end*_, *then* $\Updelta^{((D + 1)(2D+B + 1))}$ *can produce* *s*_*end*_ *via a sequence of at most* 2*D*(*D* + 1)(2*D* + *B*) *PDs*.

#### *Proof*

By definition, if UDSD $\Updelta$ contains only simple signals, each template *T* has exactly *s* + 1 configurations, where *v* is the number of domains and *s* = (*v* − 1)/2 the number of long-domains of *T*, depending on the position of the open toehold. We denote these configurations by $T_{1},\dots,T_{s + 1}$ for template *T*. Let *T*[*i*] be the domain at position *i* of *T*. Then each PD acting on the domain *i* of *T* can be expressed as follows as a chemical reaction:

$$ T[2i - 1]^{*}T[2i]^{*} + T_{i} \rightleftharpoons T[2i]^{*} T[2i + 1]^{*} + T_{i + 1} $$

where *T*[2*i* − 1]*T[2*i*]* and *T*[2*i*]*T[2*i* + 1]* are simple signal strands and where the notation * indicates that *T*[*k*]* can bind to the template at position *k*. We express all PDs of $\Updelta$ as reversible chemical reactions above and construct the initial multiset of CRN **C** as follows. Each initially unbound signal is added to the initial multiset of **C**. For each template *T*, we add molecule *T*_*i*_ corresponding to the initial configuration of *T* to the initial multiset of **C**. It is easy to see that the constructed CRN **C** exactly simulates UDSD $\Updelta$.

However, in order to apply the bound for CRNs from Sect. 2, we need to convert **C** to a tagged CRN **C′**. We express each PD acting on the domain *T*[2*i*] of *T*[*u*, *v*] as follows as a chemical reaction:

$$ \tau^{+}_{T_{i}} + T[2i - 1]^{*}T[2i]^{*} + T_{i} \rightleftharpoons \tau^{-}_{T_{i}} + T[2i]^{*}T[2i + 1]^{*} + T_{i + 1} $$

where $\tau_{T_i}^+$ and $\tau_{T_i}^-$ are unique tags of this reaction. The tagged CRN **C**′ exactly simulates CRN **C** under the assumption that there are sufficiently many tags. Assume that the template *T* has *t* copies in $\Updelta$. For one template, a single copy of each tag is enough to guarantee that the template can transform from one state to another. Therefore, we add *t* copies of tags $\tau_{T_i}^+$ and $\tau_{T_i}^-$ over all configurations *T*_*i*_ of all templates *T* to the initial multiset of tags of **C′**. This number of tags is sufficient as it allows each simulated templates to freely transform between their configurations (assuming the required simulated signal strands are available). Note that the total number of tags added for one copy of domain *T* is exactly 2*s*.

Finally, we need to determine the parameters of the constructed tagged CRN **C′**. The number of types of signal molecules which are not initially present in the initial configurations, i.e., $|S| -  |[\![\mathcal{S}_{0}]\!]|,$ is the number of types of initially bound signal strands in $\Updelta$ plus the sum of the numbers of configurations over all templates. Since the number of configurations of a template with *s* long-domains is *s* + 1, this number can be upper bounded by *B* + 2*D*. The number of tags is exactly 2*D*. The bandwidth of **C′** is 1. The theorem follows by Theorem 1.$\square$

As shown in Fig. 3, the proof of Theorem 8 will not work in the case of general signal strands, since the number of configurations of some templates can be exponential. Instead of simulating a UDSD by a tagged CRN, in Theorem 9 we will prove a bound for general (i.e., not with simple signals) UDSDs directly, reusing some ideas of the proof for tagged CRNs.

Let $\Updelta$ be a UDSD. Our goal is to show that if there is a valid sequence of PDs $\alpha=q_1,q_2,\dots,q_{|\alpha|}$ that produces a given signal *s*_*end*_ in $\Updelta ,$ for example Fig. 6, then there is a “shorter” valid sequence, γ, that produces *s*_*end*_ in a multi-copy version of $\Updelta,$ i.e., a version that initially has many copies of ${\cal  C}_{init}$. Moreover, the number of copies of ${\cal C}_{init}$ and the length of γ will be bounded by a polynomial in *B*, the number of types of signals that are initially-bound (i.e., every copy is bound) in ${\cal C}_{init}$ but are released by* α*; and *D*, the total number of long-domains of all templates. We first provide some intuition for our proof while introducing some useful definitions, and then provide the formal details in a series of claims.

To build intuition for our proof, we present three possible strategies for constructing γ. The first two strategies are flawed but provide motivation for the details of the third, correct, strategy.

**Strategy 1:** Let γ be the sequence of PDs that, starting from the initially open toehold of a template in which *s*_*end*_ is bound, “walks”, i.e., displaces the bound signals one at a time, between this open toehold and *s*_*end*_. For example, in Fig. 6, the signal t*d_3_^*^ would be used to initiate a sequence of PDs starting at the left of the second template and finally releasing *s*_*end*_ at the far right.

The γ of Strategy 1 has length at most D. However, the multiset of invader signals needed for the displacements may not be in ${\cal C}_{init}$. To overcome this problem, we need γ to release (enough copies of) each signal that is not in ${\cal C}_{init}$ but that is released by* α*. For each type *s* of signal strand in Unbound (*α*) that is bound in ${\cal  C}_{init},$ let index(*s*) be the index of the first PD of* α* that releases *s*. Let $s_1,\ldots,s_{B} (=s_{end})$ be the sequence of all such signals ordered by their indexes, i.e., ${\rm index} (s_1) < {\rm index} (s_2) < \cdots < {\rm index} (s_{B}),$ until *s*_*end*_ is produced. Let $S_{i} = \{s_{1},\dots,s_{i}\}$. Let $\alpha_i = q_1, q_2, \dots, q_{{\rm index}(s_i)}$. For example in Fig. 6, *s*_1_ is the signal *d*_1_^*^ t* and *s*_*end*_ is *d*_end_^*^ t*.

**Strategy 2:** Using Strategy 1, and taking advantage of the fact that multiple copies of ${\cal C}_{init}$ are available initially, the PDs in γ first produce (sufficiently many) copies of signal *s*_1_. This is possible because by definition of *s*_1_, there is a walk of length at most *D* to some *s*_1_ that only uses invaders in ${\cal C}_{init}$. In a similar manner, use signals in yet additional copies of ${\cal C}_{init}$ plus the newly released signals *s*_1_ to release signal *s*_2_, and so on. For example in Fig. 6, *s*_1_ is the signal *d*_1_^*^ t*, and by using multiple copies of the initial configuration, we can get copies of *s*_1_ which help us get copies of *s*_2_, etc.

The problem with this strategy is that the number of copies of ${\cal  C}_{init}$ available initially may need to be exponential in *D*, in order to release $s_{B} (=s_{end})$. Specifically, $\Uptheta(D)$ copies of ${\cal  C}_{init}$ would be needed to produce one copy of *s*_1_, e.g., in a scenario where all of the needed invaders on the walk to *s*_1_ are identical, there is only one copy of this invader in ${\cal C}_{init}$ and *s*_1_ has distance $\Uptheta(D)$ from the initially free toehold in its template. Thus we would need $\Uptheta(DX)$ copies of ${\cal C}_{init}$ to produce *X* copies of *s*_1_ using Strategy 2. By the same argument we may need $\Uptheta(DX)$ copies of $({\cal C}_{init} \cup s_1)$ to get *X* copies of *s*_2_, leading to a total of $\Uptheta(D^2X)$ copies of ${\cal C}_{init}$ to produce *X* copies of *s*_2_, and so on. To overcome this problem, we need γ to take a walk that, while still being short, is more effective in releasing needed invaders and more conservative about using them up.

**Strategy 3**: This strategy first releases a copy of *s*_1_ via a short walk* β*_1_ that uses invaders from just a single copy of ${\cal C}_{init}$ but that can also “borrow” signals from a reserve of extra copies of ${\cal C}_{init},$ as long as the signals are returned to the reserve by the end of the walk. For example in Fig. 6, we would have many copies of the initial configuration (top) which give us a reserve of signals.

**Fig. 8:**
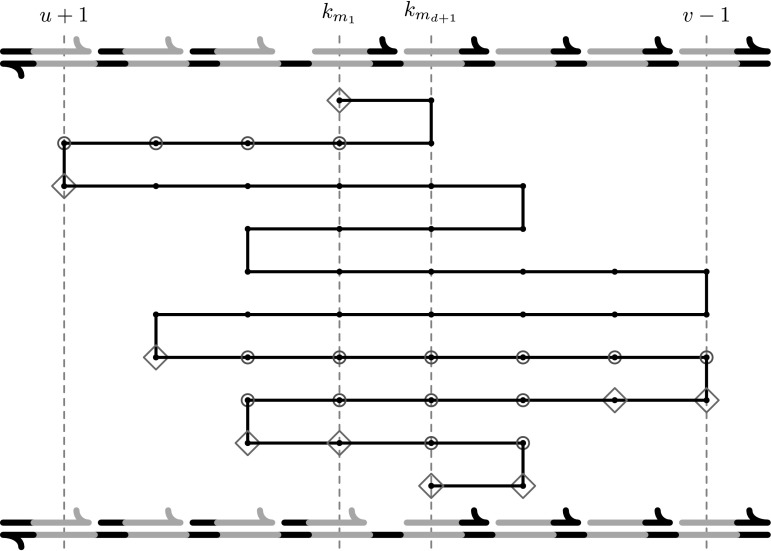
An example of construction of the* β*_*i*_(*T*) subsequence. At the* top* is the form of the initial configuration of the affected part of the template and at the* bottom* the final configuration. Each* dot* represents a PD of template subsequence* α*_*i*_(*T*), each* diamond* a marked PD and each* circle* a connector PD. The sequence of PDs* β*_*i*_(*T*) is then a subsequence of* α*_*i*_(*T*) which contains only the marked and connector PDs

We construct* β*_1_ by adapting* α*_1_ (the prefix of* α *that causes *s*_1_ to be released, see above). Note that* α*_1_ releases *s*_1_ without needing to borrow from a reserve, but may be too long for our result. In contrast,* β*_1_ will have length *O*(*D* ^2^) and at the same time, the set of initially-bound signals that are released by* β*_1_ will be the same as that of* α*_1_ and the set of signals that are finally-bound by* β*_1_ will also be the same as that of* α*_1_. In particular, all other signals, e.g., from the reserve, that may temporarily be bound during the walk taken by* β*_1_ are also released during the walk. The sequence of PDs* β*_1_ sweeps across the region traversed by* α*_1_ in a zig-zag fashion (for a specific example, see Fig. 6), so as to visit each domain for (Fig. 7) the *last* time in the same order as does* α*_1_. Figure 8 provides a general example. When visiting a domain for the last time,* β*_1_ uses the last PD of* α*_1_ that visits that domain—these PDs are called *marked* PDs in the formal description to come later. This ensures that the set of signals that are finally-bound by* β*_1_ is the same as that of* α*_1_. Also in* β*_1_, between the marked PDs, are intermediate “connector” PDs that ensure that* β*_1_ is a valid sequence of PDs. The connector PDs are also chosen so that the *first* PD of* β*_1_ that releases a signal at a given domain is the same as the first PD of* α*_1_ that releases a signal at that domain. This ensures that the set of initially-bound signals that are released by the end of* β*_1_ is the same as that of* α*_1_.

The walk* β*_1_^*X*^ can produce *X* copies of *s*_1_ using *X* copies of ${\cal C}_{init}$ plus a “reserve” of $|$ *β*_1_ $|$ copies of ${\cal C}_{init}$ that is still available at the end of the walk. In a similar fashion, copies of *s*_2_ can then be produced by consuming one additional copy of ${\cal C}_{init}$ per copy of *s*_2_, and also borrowing from the growing reserve of signals, namely multiple copies of all signals in ${\cal C}_{init} \cup \{s_1\}$. Continuing in this way, *s*_*B*_ = *s*_*end*_ can be generated from an initial number of copies of ${\cal C}_{init}$ that is bounded by a polynomial in *B* and *D*.

We now present the formal details. Let *T* be a template, and let $\alpha_{i}(T)= p_1,p_2,\dots,p_{|\alpha_{i}(T)|}$ be the template subsequence of* α*_*i*_, where *p*_*j*_ = (*I*_*j*_, *T*, *k*_*j*_, *z*_*j*_) for every $j = 1,\dots,|\alpha_{i}(T)|$. Let *u* and *v* the first and last toeholds of *T* affected by* α*_*i*_(*T*), respectively, and *d* = (*v* − *u*)/2 the number of affected long-domains in *T*. We construct a subsequence* β*_*i*_(*T*) of the PDs in* α*_*i*_(*T*). The PDs in this subsequence will be of two types, *marked* and *connector*.

#### Marked PDs

Mark the first PD *p*_1_ of* α*_*i*_(*T*), and then mark, for *each* affected long-domain in the template *T*, the *last* PD of* α*_*i*_(*T*) that binds to it. Let $$p_{m_{1}},\dots,p_{m_{d + 1}}$$ be the subsequence of all marked PDs ($$1 = m_{1} < m_{2} < \dots < m_{d + 1}$$). It is easy to see that the sequence of marked PD positions, $$k_{m_{2}},\dots,k_{m_{d}}, $$ consists of two interleaved monotonic subsequences: $$U = u + 1, u + 3, \dots,k_{m_{d + 1}} - 2$$ and $$V = v - 1, v - 3,\dots,k_{m_{d + 1}} + 2, $$ where $$k_{m_{d + 1}} $$ is the long-domain position of the last PD in* α*_*i*_(*T*). Furthermore, the marked PDs with the long-domains in the first subsequence have direction *R* and in the second subsequence direction *L*. Depending on the direction $$z_{m_{d + 1}} $$of the last marked PD, we add the long-domain position $$k_{m_{d + 1}} $$ at the end of *U* if $$z_{m_{d + 1}} $$ = *R* or at the end of *V*, if $$z_{m_{d + 1}} $$ = *L*.

#### Connector sequences

 Now, we must connect the marked PDs by introducing *connector sequences* of PDs between each consecutive pair of marked PDs with the goal being for each subsequent PD to use the toehold opened by the previous PD. Let $\bar{z}$ indicate the opposite direction from *z*.

For the connector sequence connecting $$p_{m_{1}} $$ and $$p_{m_{2}} $$, select as a connector the *first* PD in* α*_*i*_(*T*) with direction $$\bar{z}_{m_{2}}$$ that binds to each long-domain of *T* between positions $$k_{m_{2}} $$ and $$k_{m_{2}} $$ inclusive. It is easy to see that either all selected connector PDs are before $$p_{m_{2}} $$ in the sequence* α*_*i*_(*T*), or *m*_1_ = *m*_2_ and the connector sequence is empty. In the second case, $$k_{m_{1}} $$ is either *u* + 1 or *v* − 1, and there is no other PD in* α*_*i*_(*T*) with the same long-domain position.

Consider $$j = 2,\dots,d$$. Each PD of the connector sequence connecting $$p_{m_{j}} $$ to $$p_{m_{j + 1}} $$ will be between $$p_{m_{j}} $$ and $$p_{m_{j + 1}} $$ in the sequence* α*_*i*_(*T*). We will consider two cases.

1. If $$z_{m_{j}} $$ = $$z_{m_{j + 1}} $$, then no connector PDs are needed (long-domain positions $$k_{m_{j}} $$ and $$k_{m_{j + 1}} $$ are from the same subsequence—either *U* or *V*—and hence they differ by exactly 2).
2. If $$z_{m_{j}} $$≠ $$z_{m_{j + 1}} $$, then we select the connectors as follows. In the subsequence of* α*_*i*_(*T*) between PDs $$p_{m_{j}} $$ and $$p_{m_{j + 1}} $$, choose as a connector the *first* PD that binds to each position between $$k_{m_{j}} $$ and $$k_{m_{j + 1}} $$, excluding position $$k_{m_{j}} $$ and including position $$k_{m_{j + 1}} $$. Note that each PD in this connector sequence must have direction $$z_{m_{j}} $$.

The construction is illustrated in Fig. 8. The sequence* β*_*i*_(*T*) contains all the marked PDs and all the connector PDs, with distinct indices. Note that this is a subsequence of* α*_*i*_(*T*) since for every $$j = 1,\dots,d, $$ the connector sequence connecting $$p_{m_{j}}$$ to $$p_{m_{j + 1}} $$ contains only PDs between between $$p_{m_{j}} $$ and $$p_{m_{j + 1}} $$. Finally, we define* β*_*i*_ as a concatenation of* β*_*i*_(*T*)’s over all templates *T* in $$\mathcal{C}_{init}$$.

We next state and prove a sequence of claims that we use to prove our main result.

##### **Claim 2**

*The first PD in the sequence* *β*_*i*_(*T*)* can use the initially open toehold. Every other PD in the sequence can use the toehold opened by the previous PD in the sequence.*

##### *Proof*

The first part of the claim is straightforward since the first PD of* β*_*i*_(*T*), i.e., $$p_{m_{1}} $$, is also the first PD of* α*_*i*_(*T*).

For the second part of the claim, first, we note that each connector sequence connecting two consecutive marked PDs consists of PDs with the same direction such that for all two consecutive PDs in the connector sequence, their long-domain positions differ by the same value in {−2,2}. Therefore, all but the first PD in the connector sequence uses a toehold open by the previous PD in the sequence. It is enough to show that this condition is satisfied also

- between the first PD of a connector sequence and the preceding marked PD; and
- between the last PD of a connector sequence and the following marked PD,

for each non-empty connector sequence. In the case that some connector sequence is empty, we need to check that the condition is satisfied between the marked PDs preceding and following the empty connector sequence. Consider the later case first: assume that the connector sequence connecting $$p_{m_{j}} $$ to $$p_{m_{j + 1}} $$ is empty. Then $$k_{m_{j}} $$ and $$k_{m_{j + 1}} $$ belong to the same monotonic subsequence (either *U* or *V*), i.e., $$k_{m_{j + 1}} $$ = $$k_{m_{j}} $$ ± 2, and hence, $$p_{m_{j + 1}} $$ uses the toehold opened by $$p_{m_{j}} $$.

Consider the connector sequence connecting $$p_{m_{1}} $$ to $$p_{m_{2}} $$. If *m*_1_ = *m*_2_, both conditions are trivially satisfied. Otherwise, PD $$p_{m_{1}} $$ may or may not be in this connector sequence. If $$p_{m_{1}} $$ is a connector, the first PD of the connector sequence is $$p_{m_{1}} $$, hence condition (a) is trivially satisfied. If PD $$p_{m_{1}} $$ is not a connector then $$z_{m_{1}} $$ = $$z_{m_{2}} $$ and either the connector sequence is empty, or the long-domain position of the first PD of the connector sequence is $$k_{m_{1}} $$, that is, the first PD of the connector sequence uses the toehold opened by $$p_{m_{1}} $$, condition (a) holds. The long-domain position of the last PD of the non-empty connector sequence is $$k_{m_{2}} $$ and the direction of the last PD is $$\bar z_{m_{2}}, $$ hence condition (b) is satisfied.

Next, consider the connector sequence connecting $$p_{m_{j}} $$ to $$p_{m_{j + 1}} $$, for $$j = 2,\dots,d$$.

1. If $z_{m_{j}} = z_{m_{j + 1}}$, then the connector sequence is empty.
2. If $$z_{m_{j}}\ne z_{m_{j + 1}}$$, then the long-domain position of the first PD in the connector sequence is $$k_{m_{j}} $$ + 2 if $$z_{m_{j}} $$ = *R* and $$k_{m_{j}} $$ − 2 if $$z_{m_{j}} $$ = *L*. In either case, the condition (a) is satisfied. Furthermore, the long-domain position of the last PD of the connector sequence is $$k_{m_{j + 1}} $$ and its direction is $$\bar z_{m_{j + 1}}, $$ hence, condition (b) is satisfied. $$\square$$

##### **Claim 3**

*The length of* *β*_*i*_(*T*)* is at most (δ + 1)(δ + 2)/2, where δ is the number of long-domains in* *T*.

##### *Proof*

Let *u* and *v* be the first and last affected toeholds of *T* by* β*_*i*_(*T*) and *d* = (*v* − *u*)/2 ≤ δ the number of affected long-domains. The number of marked PDs is *d* + 1. The first connector sequence has at most *d* PDs. For each $$j = 2,\dots,d, $$ consider the connector sequence connecting marked PDs $$p_{m_{j}} $$ and $$p_{m_{j + 1}} $$. If $$z_{m_{j}} = z_{m_{j + 1}}$$, the sequence is empty. Otherwise, the number of PDs in the sequence is at most $$|k_{m_{j + 1}} - k_{m_{j}}|/2$\, {\text{and}}\,$k_{m_{j}}$$ and $$z_{m_{d}} $$ and $$k_{m_{j + 1}} $$ belong to different monotonic subsequences of positions. Without loss of generality, assume $$k_{m_{j}} $$ is at index *r* in *U* and $$k_{m_{j + 1}} $$ is at index *r*′ in *V*. Since each marked PD advances by one element in exactly one of the sequences *U* and *V*, we have *r* + *r*′ = *j*, and therefore $$|k_{m_{j + 1}} - k_{m_{j}}|/2 = |[v - (2r' - 1)] - [u + (2r - 1)]|/2 = |v - u - 2j + 1|/2 = d - j + 1$$ (the last equality follows since *j* ≤ *d*). Hence, the number of connector PDs is at most *d* + ∑_*j*=2_^*d*^ (*d* − *j* + 1) = *d*(*d* + 1)/2 and the total number of PDs in* β*_*i*_(*T*) as at most (*d* + 1)(*d* + 2)/2 ≤ (δ + 1)(δ + 2)/2. $$\square$$

##### **Claim 4**

*The length of* *β*_*i*_ *is at most* (*D* + 1)(*D* *+ 2)/2 and thus* $|$Invaders(*β*_*i*_)$|$ ≤ (*D* + 1)(*D + 2)/2. Also,* Invaders(*β*_*i*_)* contains only types of unbound strands of* ${\cal C}_{init}$ *or strand types in* $S_{i - 1} = \{s_1, \ldots, s_{i-1}\}$.

##### *Proof*

By Claim 3, for each template *T*, the number of PDs of* β*_*i*_(*T*) is at most (δ + 1)(δ + 2)/2, where δ is the number of long-domains in *T*. Summing through all domains, we obtain that the length of* β*_*i*_ is at most (*D* + 1)(*D* + 2)/2.

By definition of* α*_*i*_, Invaders(*α*_*i*_) contains only types of unbound strands of ${\cal C}_{init}$ or strand types in $\{s_1, \ldots, s_{i-1}\}$. Since* β*_*i*_ is a subsequence of* α*_*i*_, it must also be that Invaders(*β*_*i*_) also contains only types of unbound strands of ${\cal C}_{init}$ or strand types in *S*_*i*−1_. $\square$

##### **Claim 5**

*β*_*i*_ *is valid with respect to
*

$$ {\cal C}_{init} \cup (D + 1)(D + 2)/2 \cdot ({\mathcal{C}}_{init} \cup S_{i - 1}). $$

Moreover,

$$ (D + 1)(D + 2)/2 \cdot ({\cal C}_{init} \cup S_{i - 1}) \subseteq {\rm Unbound}(\beta_i, {\cal C}_{init} \cup (D + 1)(D + 2)/2 \cdot ({\cal C}_{init} \cup S_{i - 1})). $$

##### *Proof*

Let $\beta_i = p_1',p_2',\ldots,p_{|\beta_i|}'$. To prove the first part of the claim, we need to show that there is a sequence ${\cal C}_1, {\cal C}_2, \ldots {\cal C}_{|\beta_i|+1}$ of configurations with ${\cal C}_1 = {\cal C}_{init} \cup (D + 1)(D + 2)/2 \cdot ({\cal C}_{init} \cup S_{i-1})$ such that for all *j*, 1 ≤ *j* ≤ |*β*_*i*_|, *p*_*j*_′ is applicable to ${\cal C}_j$ and induces ${\cal C}_{j+1}$ from ${\cal C}_j$. We can prove this by induction on *j*. The base case when *j* = 1 is trivial. Suppose that *j* > 1, and that *p*′_*j*−1_ is applicable to ${\cal C}_{j-1}$ and induces ${\cal C}_{j}$ from ${\cal C}_{j-1}$. Let *p*_*j*_′ = (*I*, *T*, *k*, *z*). Since (*I*, *T*, *k*, *z*) is also a PD of* α*_*i*_ and* α*_*i*_ is valid, it is straightforward to check that condition 1 of the definition of “applicable” must hold. Condition 2 also holds because *j* ≤ (*D* + 1)(*D* + 2)/2 and there are (*D* + 1)(*D* + 2)/2 copies of all unbound signals used by* β*_*i*_ initially present in $(D + 1)(D + 2)/2 \cdot (\mathcal{C}_{init} \cup S_{i - 1})$. So, we assume that *z* = *R* and show that condition 3 holds (the argument is similar when *z* = *L*). Condition 3a and 3b also follow simply from the fact that (*I*, *T*, *k*, *z*) is a PD of* α*.

Condition 3c, that the releasee is not bound to any domain except the neighboring toehold, must be true because (*I*, *T*, *k*, *z*) is a PD of* α*. The condition 3d follows by Claim 2.

The final multiset of signals after executing PDs in* α*_*i*_ on ${\cal C}_{init}$ is the multiset consisting of all signals of ${\cal C}_{init}$ plus all signals initially bound to domains that appear in PDs of* α*_*i*_ minus all signals that are finally bound to domains that appear in PDs of* α*_*i*_. By construction, PDs in* β*_*i*_ operate on exactly the same set of long-domains as PDs in* α*_*i*_ and the last PD applied to each long-domain of* α*_*i*_ is exactly the same as those of* β*_*i*_. Therefore, no matter whether we execute PDs in* α*_*i*_ or PDs in* β*_*i*_, exactly the same set of signals are released and bound, and hence, the final multiset of unbound signals is the same as well. It follows that $\square$

$$ (D + 1)(D + 2)/2 \cdot ({\cal C}_{init} \cup S_{i - 1}) \subseteq {\rm Unbound}(\beta_i,{\cal C}_{init} \cup (D + 1)(D + 2)/2 \cdot ({\cal C}_{init} \cup S_{i - 1})). $$

##### **Claim 6**

*Let* *β*_*i*_^(*D*+1)(*D+2)/2*^ denote the sequence * β*_*i*_ *concatenated* (*D* + 1)(*D* *+ 2)/2 times, modified just so that the PDs of each copy refer to templates of different copies of* $(D + 1)(D + 2)/2 \cdot {\cal C}_{init}$.* Then* *β*_*i*_^(*D*+1)(*D+2)/2*^ is valid with respect to the configuration

$$ (D + 1)(D + 2)/2 \cdot {\cal C}_{init}\,\cup (D + 1)(D + 2)/2 \cdot ({\mathcal{C}}_{init} \cup S_{i - 1}). $$

Moreover,

1 $$ (D + 1)(D + 2)/2 \cdot ({\cal C}_{init} \cup S_{i}) \subseteq {\rm Unbound}(\beta_i^{(D + 1)(D + 2)/2}, (D + 1)(D + 2)/2 \cdot {\cal C}_{init} \cup (D + 1)(D + 2)/2 \cdot ({\cal C}_{init} \cup S_{i - 1})) $$

##### *Proof*

By Claim 5,* β*_*i*_ is valid with respect to ${\cal C}_{init}\cup (D + 1)(D + 2)/2 \cdot ({\cal C}_{init} \cup S_{i - 1})$. Moreover, the final multiset of signals is the same as if we were to execute PDs in* α*_*i*_ on ${\cal C}_{init}$ and then add $(D + 1)(D + 2)/2 \cdot ({\cal C}_{init} \cup S_{i - 1})$. Thus, if we repeat* β*_*i*_ (*D* + 1)(*D* + 2)/2 times and execute each* β*_*i*_ on a different copy of ${\cal C}_{init},$ we will still have at least (*D* + 1)(*D* + 2)/2 copies of signals in ${\cal C} \cup S_{i - 1}$ plus (*D* + 1)(*D* + 2)/2 copies of *s*_*i*_.$\square$

The proof of our main technical result, Theorem 9, follows from the preceding claim.

##### **Theorem 9**

*Let* $\Updelta$ *be a UDSD with* *B* *types of initially bound signal strands and let* *D* *be the total number of long-domains of all templates*. *If* $\Updelta$ *can produce* *s*_*end*_, *then* $\Updelta^{((D + 1)(D + 2)(B + 1)/2)}$ *can produce* *s*_*end*_ *via a sequence of at most* (*D* + 1)^2^(*D* + 2)^2^ *B*/4 *PDs*.

##### *Proof*

Let* α*, *α*_*i*_ and* β*_*i*_, 1 ≤ *i* ≤ *B* be defined as above. Let γ be the sequence of PDs obtained by concatenating (*D* + 1)(*D* + 2)/2 copies of sequence* β*_1_ followed by (*D* + 1)(*D* + 2)/2 copies of* β*_2_ and so on up to (*D* + 1)(*D* + 2)/2 copies of* β*_*B*_, and modifying each copy just so that the PDs of each copy refer to templates of different copies of $(D + 1)(D + 2)B/2 \cdot {\cal C}_{init}$.

By applying Claim 6, we will show by induction on *i* that

$$ \beta_1^{(D + 1)(D + 2)/2} \beta_2^{(D + 1)(D + 2)/2} \ldots \beta_i^{(D + 1)(D + 2)/2} $$

is valid with respect to $(D + 1)(D + 2)(i + 1)/2 \cdot {\cal C}_{init}$ and that

2 $$ (D + 1)(D + 2)/2 \cdot ({\mathcal{C}}_{init} \cup S_{i}) \subseteq {\rm Unbound}(\beta_1^{(D + 1)(D + 2)/2} \beta_2^{(D + 1)(D + 2)/2} \ldots \beta_i^{(D + 1)(D + 2)/2}, (D + 1)(D + 2)(i + 1)/2 \cdot {\cal C}_{init}). $$

It follows that γ is valid with respect to $(D + 1)(D + 2)(B + 1)/2 \cdot {\cal C}_{init}$ and that γ produces *s*_*end*_ (=*s*_*B*_). The base case is when *i* = 1. It follows directly from Claim 6 that* β*_1_^(*D*+1)(*D*+2)/2^ is valid with respect to the configuration $(D + 1)(D + 2) \cdot {\cal C}_{init}$ and that $(D + 1)(D + 2)/2 \cdot (\mathcal{C}_{init} \cup S_{1}) \subseteq {\rm Unbound}(\beta_1^{(D + 1)(D + 2)/2},(D + 1)(D + 2)\cdot {\cal C}_{init})$. The induction hypothesis is that $\beta_1^{(D + 1)(D + 2)/2} \beta_2^{(D + 1)(D + 2)/2} \ldots \beta_{i-1}^{(D + 1)(D + 2)/2}$ is valid with respect to $(D + 1)(D + 2)(i+1)/2 \cdot {\cal C}_{init}$ and that

3 $$ (D + 1)(D + 2)/2 \cdot ({\mathcal{C}}_{init} \cup S_{i - 1}) \subseteq {\rm Unbound}(\beta_1^{(D + 1)(D + 2)/2} \beta_2^{(D + 1)(D + 2)/2} \ldots \beta_{i-1}^{(D + 1)(D + 2)/2}, (D + 1)(D + 2)(i + 1)/2 \cdot {\cal C}_{init}) $$

The induction step then follows easily from Claim 6 because after the PDs in

$$ \beta_1^{(D + 1)(D + 2)/2} \beta_2^{(D + 1)(D + 2)/2} \ldots \beta_{i-1}^{(D + 1)(D + 2)/2} $$

have been applied on $(D + 1)(D + 2)(i + 1)/2 \cdot {\cal C}_{init},$ the resulting configuration includes both $(D + 1)(D + 2)/2 \cdot (\mathcal{C}_{init} \cup S_{i - 1})$ (by the induction hypothesis) and (*D* + 1)(*D* + 2)/2 additional copies of ${\cal C}_{init}$ needed for sequence* β*_*i*_^(*D*+1)(*D*+2)/2^. Also, each of the (*D* + 1)(*D* + 2)/2* β*_*i*_ sequences produces a single *s*_*i*_ that remains unbound when the remaining* β*_*i*_’s are applied.□

Finally, we restate Theorem 9 for copy-tolerant UDSDs. We say that a UDSD is *x*-copy-tolerant if the length of the shortest PD sequence that produces any signal strand *s* in $\Updelta$ and in $\Updelta^{(x)}$ is the same. A UDSD is copy-tolerant if it is *x*-copy-tolerant for all *x*.

##### **Theorem 10**

*Let* $\Updelta$ *be a UDSD with* *B* *types of initially bound signal strands and let* *D* *be the total number of long-domains of all templates. If* $\Updelta$ *can produce* *s*_*end*_ *and* $\Updelta$ *is* (*D* + 1)(*D* + 2)(*B* + 1)/2-*copy tolerant*, *then* $\Updelta$ *can produce* *s*_*end*_ *via a sequence of at most* (*D* + 1)^2^(*D* + 2)^2^ *B*/4 *PDs*.

#### Concatenated templates

 The result above may seem to be limited, due to the definition of templates beginning and ending with toehold domains and consisting of alternating toehold and long-domains. Let a *generalized template* consist of several templates concatenated together. In fact, the result as stated in Theorem 9 also applies to generalized templates. This is because a UDSD with generalized templates can be simulated w.l.o.g. by a UDSD with a sufficient number of templates. This makes our result more general.

#### Irreversible reactions

 If irreversible reactions are considered, then we must allow for there to be no toehold to either the left or right of a long-domain on the template. If we are to keep the condition that every PD has a releasee, then we must allow for some releasee to contain only the long-domain, rather than the toehold. This complicates the proofs, because the current development maintains that at any time there is only one open toehold in each template. In order to both allow irreversible reactions and use the current proofs, we must require that in ${\cal C}_{init}$ one-domain releasees only appear where there is no toehold to either the right or the left. Because we find this restriction somewhat artificial, we conjecture that there is some generalization of these proof that allows for irreversible reactions.

## Conclusions and open questions

In this paper, we have considered three models of biomolecular programs, namely tagged CRNs, DSDs, and DSDs with simple signals. We have shown that, when multiple copies of all initial molecules are present, such programs fail to work correctly if the number of reactions of the program is sufficiently large relative to the volume of initial reagents. A natural question is: how do these models relate to each other, in the sense that one can be simulated by another? Soloveichik et al. showed how CRNs (and thus also tagged CRNs) could be simulated by DSDs, in the sense that CRN species are mapped to DSD signals, CRN reactions can be simulated by a cascade of DSD strand displacements, and the dynamical properties of the CRN are reproduced. As a consequence, programs specified as CRNs can be compiled into real, DNA-based chemical systems and there are several examples to date. We are not aware of general methods for simulating DSDs by CRNs. Such simulations are possible in principle, for example by mapping each multi-stranded complex that could arise in a configuration of the DSD to be simulated, to distinct abstract species of the simulating CRN. However, the number of species could be exponential in the size of the DSD, and it’s not clear what purpose such a simulation would serve.

There are many open questions about the potential for CRNs and DSDs to be correct in the multi-copy setting. First, can our reachability upper bound results be strengthened? There are two possible ways to strengthen our result for CRNs (Theorem 2): either by reducing the length of the shortest computation needed to produce *s*_*end*_ or to show that the system is not *x*-copy tolerant for some ${x<|{S}|\!{b_{C}}\,{\left({T_{C}}/2 + 1\right)}}$. Similarly, there are two ways to strengthen the reachability upper bounds for DSDs.

Also, can our result on DSDs be extended to DSDs with more complex primitives, such as cooperative strand displacement (Zhang 2011) or irreversible reactions? What if long-domains can form intra-molecular bonds, e.g., forming hairpins, in addition to inter-molecular bonds?

This paper considers only reachability bounds, i.e., bounds on the number of reactions (steps) needed to reach (produce) a given product. However, real CRNs behave stochastically, with rates that depend on relative quantities of species. It is plausible that the lack of robustness implied by our theorems, i.e., errors that occur in the multi-copy setting in CRNs that fail to satisfy the conditions of the theorem, would be very unlikely to occur in some CRNs and thus would not be an issue in a real system. Analyses of robustness of CRNs under stochastic assumptions, perhaps computing expected hitting times, would help us better understand the degree to which robustness issues are a problem.

## Footnotes

1 Volume refers to the physical volume of all the molecules. The initial volume can be approximated by the total number of all types of reagents in the initial configurations.

2 A graph that can be drawn in a way that all vertices lie on a circle and edges lie inside the circle and do not cross. In graph theory, the formal equivalent of circular graphs are outerplanar graphs.
